# Analysis of the senescence‐associated cell surfaceome reveals potential senotherapeutic targets

**DOI:** 10.1111/acel.14312

**Published:** 2024-09-03

**Authors:** Yushuang Deng, Ting Liu, Enzo Scifo, Tao Li, Kan Xie, Bernd Taschler, Sarah Morsy, Kristina Schaaf, Armin Ehninger, Daniele Bano, Dan Ehninger

**Affiliations:** ^1^ Translational Biogerontology Lab German Center for Neurodegenerative Diseases (DZNE) Bonn Germany; ^2^ Department of Neurodegenerative Disease and Geriatric Psychiatry/Neurology University of Bonn Medical Center Bonn Germany; ^3^ Big Data Institute University of Oxford Oxford UK; ^4^ AvenCell Europe GmbH Dresden Germany; ^5^ Aging and Neurodegeneration Lab German Center for Neurodegenerative Diseases (DZNE) Bonn Germany

**Keywords:** aging, cell surface proteins, cellular senescence, mass spectrometry, senotherapeutics

## Abstract

The accumulation of senescent cells is thought to play a crucial role in aging‐associated physiological decline and the pathogenesis of various age‐related pathologies. Targeting senescence‐associated cell surface molecules through immunotherapy emerges as a promising avenue for the selective removal of these cells. Despite its potential, a thorough characterization of senescence‐specific surface proteins remains to be achieved. Our study addresses this gap by conducting an extensive analysis of the cell surface proteome, or “surfaceome”, in senescent cells, spanning various senescence induction regimes and encompassing both murine and human cell types. Utilizing quantitative mass spectrometry, we investigated enriched cell surface proteins across eight distinct models of senescence. Our results uncover significant changes in surfaceome expression profiles during senescence, highlighting extensive modifications in cell mechanics and extracellular matrix remodeling. Our research also reveals substantive heterogeneity of senescence, predominantly influenced by cell type and senescence inducer. A key discovery of our study is the identification of four unique cell surface proteins with extracellular epitopes. These proteins are expressed in senescent cells, absent or present at low levels in their proliferating counterparts, and notably upregulated in tissues from aged mice and an Alzheimer's disease mouse model. These proteins stand out as promising candidates for senotherapeutic targeting, offering potential pathways for the detection and strategic targeting of senescent cell populations in aging and age‐related diseases.

## Graphical abstract

We employed label‐free quantitative mass spectrometry to comprehensively investigate the cell surface proteome across eight distinct senescence models. Our findings highlighted extensive modifications in cell mechanics and extracellular matrix remodeling during senescence. Additionally, we uncovered significant heterogeneity in the senescence surface proteome, predominantly influenced by cell type and senescence inducer. A key discovery of our study is the identification of four unique cell surface proteins with extracellular epitopes as potential senotherapeutic targets.

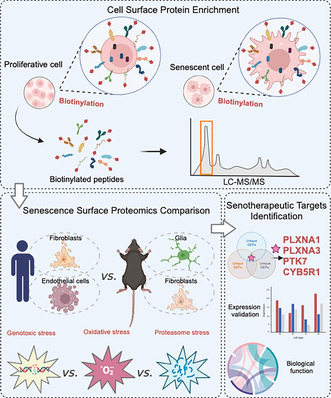

**Abbreviations**
ACN: acenonitrile
AD: Alzheimer's disease
ADCs: antibody‐drug conjugates
AGA: n(4)‐(beta‐N‐acetylglucosaminyl)‐L‐asparaginase
ANOVA: analysis of variance
AST: mouse astrocytes
BSA: bovine serum albumin
CA: cornu ammonis
CAR: chimeric antigen receptor
CCL: chemokine (C‐C motif) ligand
CS: cellular senescence
CXCL: chemokine (C‐X‐C motif) ligand
CYB5R1: cytochrome b5 reductase 1
DAPI: 4',6‐diamidino‐2‐phenylindole
DEPs: differentially expressed proteins
DG: dentate gyrus
DMEM: dulbecco's modified eagle's medium
DMSO: dimethyl sulfoxide
DPBS: dulbecco's phosphate‐buffered saline
DPP4: dipeptidyl peptidase 4
ECM: extracellular matrix
EPO: epoxomicin
ETO: etoposide
EVs: extracellular vesicles
FA: formic acid
FASP: filter‐aided‐sample‐preparation
FBS: fetal bovine serum
FC: fold change
FDR: false discovery rate
FMR1: fragile X messenger ribonucleoprotein 1
GFAP: glial fibrillary acidic protein
GO: gene ontology
GPNMB: glycoprotein nonmetastatic melanoma protein B
GPX7: glutathione peroxidase 7
HBSS: hanks' balanced salt solution
H_2_O_2_
: hydrogen peroxide
HPM: human proteome map
HUVECs: human umbilical vein endothelial cells
IBA1: ionized calcium binding adaptor molecule 1
ICAM1: intercellular adhesion molecule‐1
IL: interleukin
LC‐MS/MS: liquid chromatography‐tandem mass spectrometry
LORs: log‐odds ratios
L‐R: ligand‐receptor
MEFs: mouse embryonic fibroblasts
MKI67: marker of proliferation Ki‐67
MMP: matrix metallopeptidase
MS: mass spectrometry
NHLFs: normal human lung fibroblasts
PCA: principal component analysis
PCCs: pearson correlation coefficients
PFA: paraformaldehyde
PLXNA: plexin A
PSMs: peptide spectral matches
PTK7: protein tyrosine kinase 7
qPCR: quantitative real‐time PCR
ROS: reactive oxygen species
RS: replicative senescence
RSLC: rapid separation liquid chromatography
RTK: receptor tyrosine kinase
SASP: senescence‐associated secretory phenotype
SA‐β‐gal: senescence‐associated beta‐galactosidase
SCAMP4: secretory carrier membrane protein 4
scRNA‐seq: single‐cell RNA sequencing
SEN/CTRL: senescence/control
TBS: tris‐buffered saline
TMS: tabula muris senis
TNF‐α: tumor necrosis factor alpha
WT: wild type

## 1 INTRODUCTION

Cellular senescence (CS) is a state of irreversible cell‐cycle arrest, triggered by a wide range of intrinsic and extrinsic cellular stressors, including DNA damage, hypoxia, mitochondrial dysfunction, and oncogene activation (Di Micco et al., 2021; Gorgoulis et al., 2019; Hayflick & Moorhead, 1961; Kuilman et al., 2010). One of the well‐known features of CS is the over‐activation of the p16^ink4a^ and/or p53‐p21^Cip1/Waf1^ pathways, which inhibit cyclin‐dependent kinases to block cell cycle progression (Hernandez‐Segura et al., 2018; Purvis et al., 2012; Sherr et al., 2016). An additional notable trait exhibited by senescent cells is the senescence‐associated secretory phenotype (SASP), encompassing the secretion into the extracellular space of a broad spectrum of proteins, such as chemokines, growth factors, proinflammatory cytokines, and matrix metalloproteases. This secretome, specific to senescent cells, can impact neighboring cells, induce a chronic inflammatory milieu, and is ultimately thought to contribute to marked tissue dysfunction (Coppé et al., 2010; Tchkonia et al., 2013).

CS exerts a range of effects on both physiological and pathological processes (Muñoz‐Espín & Serrano, 2014). In young organisms, CS aids in wound healing and prevents tumorigenesis (He & Sharpless, 2017). However, senescent cells accumulate in multiple tissues with age and can exacerbate age‐associated tissue changes, such as chronic inflammation, cardiac insufficiency, and others (Childs et al., 2015; He & Sharpless, 2017; Kowald et al., 2020). Indeed, studies employing genetic tools, such as the INK/ATTAC and p16‐3MR mouse models (Baker et al., 2011; Demaria et al., 2014; Hashimoto et al., 2016), or pharmacological interventions, known as senolytics (inducing selective CS apoptosis) and senomorphics (suppressing SASP) (Zhang et al., 2023), have demonstrated the potential of senescent cell ablation to improve various age‐related morbidities, including neurodegenerative diseases, such as Alzheimer's disease (AD) (Bussian et al., 2018; P. Zhang et al., 2019), cardiovascular pathologies (Childs et al., 2016), cancers (Baker et al., 2016), chronic pulmonary diseases (Schafer et al., 2017), and osteoarthritis (Jeon et al., 2017) in mice. Despite the significant senolytic efficacy established by these approaches, important challenges remain. These include the lack of translatability of genetic tools, as well as the limited specificity and undesired side effects of available senolytics/senomorphics. Recently, immunotherapies targeting senescent cell surface molecules, such as senolytic Chimeric Antigen Receptor (CAR)‐T cells, have been shown to counter a range of age‐related physiological alterations in mice (Amor et al., 2020; Qu et al., 2020; Suda et al., 2021; Yang et al., 2023). Conceptually, this approach aims to take advantage of surface markers specifically expressed in CS, thereby promising to offer heightened precision for in vivo senescent cell elimination while plausibly limiting the side effects on the normal, non‐senescent cells. However, the prerequisite for this targeted removal of senescent cells lies in unequivocally detecting and identifying senescent cells in tissues based on unique and senescence‐specific cell surface markers.

Although several markers, such as upregulated p16 and/or p21, increased lysosomal senescence‐associated beta‐galactosidase (SA‐β‐gal) activity at pH 6, accumulated lipofuscin, and various chromatin features, have been commonly reported and used in CS studies (Hernandez‐Segura et al., 2018), their individual utility for conclusive identification of senescent cells is limited due to the heterogeneity of CS and the lack of specificity of each of these individual markers. Advances in unbiased RNA‐sequencing‐based transcriptome analyses (Casella et al., 2019; Hernandez‐Segura et al., 2017) and mass spectrometry (MS)‐based proteome analyses (Althubiti et al., 2014; Basisty et al., 2020) have substantially expanded our understanding of CS expression profiles. However, the large‐scale identification of surface proteins associated with CS remains constrained by their low solubility and abundance. Recent efforts focusing on CS surface markers have identified several molecules, including intercellular adhesion molecule‐1 (ICAM1), dipeptidyl peptidase 4 (DPP4), and secretory carrier membrane protein 4 (SCAMP4), through screening of cell surface‐associated proteins preferentially expressed in human replicative senescent (RS) fibroblasts (K. M. Kim et al., 2018, 2017; Mrazkova et al., 2018). Most recently, unbiased proteomic screening of differentially expressed plasma membrane proteins in therapy‐induced senescence of human SK‐MEL‐103 melanoma cells identified an upregulation of PD‐L2 in various cancer cells which may play a crucial role in evading immune surveillance (Chaib et al., 2024). Despite these important advancements in the field of the senescence surfaceome, translating these findings into effective senotherapeutic strategies is still challenging. A key hurdle may be attributed to the limited cellular origins, senescence stimuli, and species upon which senescent cell models were established in previous studies.

In this study, we sought to characterize the spectrum of cell‐surface proteins in senescent cells induced by diverse stimuli across various murine and human cell types of both fetal and adult origins. The aim was to identify potential cell surface candidates for senotherapy. We utilized a cell‐surface biotinylation method to enrich cell surface proteins and conducted label‐free quantitative MS‐based proteomics analysis on normal human lung fibroblasts (NHLFs), human umbilical vein endothelial cells (HUVECs), mouse embryonic fibroblasts (MEFs), and mouse astrocytes (AST) subjected to genotoxic, oxidative or proteasome stress. We identified a set of CS surface markers and validated their enrichment in various tissues of naturally aged mice, as well as in the brains of APP/PS1 mice, an AD mouse model featuring senescence accumulation in the Aβ plaque environment (P. Zhang et al., 2019). This study provides foundational work for potential future applications aimed at detecting and targeting senescent cells during natural aging and age‐related diseases in vivo.

## 2 MATERIALS AND METHODS

### 2.1 Mice

C57BL/6J wild‐type (strain #000664) and APP/PS1 (B6.Cg‐Tg(APPswe, PSEN1dE9)85Dbo/Mmjax, strain #034832) mice used in this study were purchased from The Jackson Laboratory. All animals were group housed (up to five animals per cage) in individually ventilated cages under specific pathogen‐free conditions. Mice were kept on a 12:12 h light–dark cycle at a constant temperature of 22°C and 55% humidity. Food and water were supplied ad libitum. In accordance with the German Animal Welfare Act, the present study was approved by the “Landesamt für Natur, Umwelt und Verbraucherschutz Nordrhein‐Westfalen” (Recklinghausen, Germany). Local and federal animal welfare regulations were followed. Adult animals were sacrificed by an overdose of isoflurane. For quantitative real‐time PCR (qPCR) and Western blot analysis, mouse tissues were snap‐frozen in liquid nitrogen, and stored at −80°C until further use. For histological staining, mouse brains and testes were fixed in 4% paraformaldehyde (PFA)/PBS (Santa Cruz Biotechnology, #sc‐281692) at 4°C overnight after perfusion with ice‐cold PBS. On the following day, the fixed tissue was rinsed three times in PBS and subsequently maintained in PBS containing 0.05% NaN3 at 4°C until use.

### 2.2 Cell culture

All cells were cultured at 37°C in a humidified 5% CO_2_ atmosphere. Primary HUVECs (Lonza, #C2517A) were cultured in EBM‐2 Basal Medium (Lonza, #CC‐3156) with EGM‐2 SingleQuots supplements (Lonza, #CC‐4176). Primary NHLFs (Lonza, #CC‐2512) were cultured in FBM Fibroblast Growth Basal Medium (Lonza, #CC‐3131) with FGM‐2 SingleQuot supplements and Growth Factors (Lonza, #CC‐4126). Primary astrocytes from mouse forebrains were prepared as previously described (Schildge et al., 2013). In brief, forebrains of P2‐P4 wild‐type C57BL/6J mouse pups were isolated and minced after the removal of the meninges. The brains were washed with Hanks' Balanced Salt Solution (HBSS, Thermo Fisher Scientific) and incubated with trypsin (Thermo Fisher Scientific) and DNase I (Roche) for 10 min at 37°C. Then the mixed brain cells were plated in T75 flasks in Dulbecco's modified Eagle's medium (DMEM, Thermo Fisher Scientific) supplemented with 10% fetal bovine serum (FBS, Thermo Fisher Scientific) and 1% penicillin–streptomycin (Thermo Fisher Scientific), then incubated to confluency (7–10 days). Microglia were first removed by shaking the T75 flask at 180 rpm for 30 min at 37°C on an orbital shaker and oligodendrocyte precursor cells were removed afterwards by shaking the flask at 240 rpm for 6 h at 37°C, followed by tapping against the flask by hand for 1 min. Astrocytes were then cultured in a fresh complete medium. Primary MEFs were isolated from E13.5 wild‐type C57BL/6J embryos as previously described (Jain et al., 2014). In brief, the embryos were isolated and minced after the removal of visceral organs. Cells were digested by trypsinization and cultured in DMEM supplemented with 10% FBS and 1% penicillin–streptomycin. For each biologically independent experiment, primary cell cultures were prepared independently. Cells were not pooled.

### 2.3 Senescence induction

The applied dosages of CS inducers as well as treatment duration were optimized for the respective senescent cell model included in the current study. For genotoxic stress‐induced senescence, P1‐P3 NHLFs, and MEFs were treated with 5 μM and 10 μM Etoposide (ETO; Merck) for 48 h. Subsequently, cells were washed with Dulbecco's phosphate‐buffered saline (DPBS; Thermo Fisher Scientific) and harvested 5 days later. P1‐P2 mouse astrocytes were treated with 5 μM ETO for 24 h, then washed and harvested 2 days later. For oxidative stress‐induced senescence, P1‐P3 HUVECs and NHLFs were treated with 100 μM hydrogen peroxide (H_2_O_2_; Merck) for 5 days, then washed and cultured in a complete medium for an additional 2 days. P1‐P2 MEFs and mouse astrocytes were treated with 200 μM H_2_O_2_ for 2 h, then washed with DPBS. H_2_O_2_‐treated MEFs were cultured in a complete medium for an additional 7 days and H_2_O_2_‐treated mouse astrocytes were cultured in a complete medium for additional 3 days. For proteasome stress‐induced senescence, P1‐P2 mouse astrocytes were incubated for 6 days in a complete medium in the presence of 5 nM Epoxomicin (EPO; MedChemExpress). P1‐P3 proliferating cells treated with 0.1% dimethyl sulfoxide (DMSO; Merck) served as vehicle controls. Cells used for all conditions were at about 60% confluency before treatments.

### 2.4 RNA extraction, reverse transcription, and quantitative real‐time PCR

Total RNA was isolated using RNeasy Mini Kit (Qiagen, #74104) according to the manufacturer's protocol. cDNA was synthesized using High‐Capacity cDNA Reverse Transcription Kit (Thermo Fisher Scientific). qPCR was then performed with PowerUP SYBR Green Master Mix (Thermo Fisher Scientific) on a QuantStudio6 Real‐Time PCR System (Thermo Fisher Scientific). Forward and reverse primers used are listed in Table S1.

### 2.5 Western blot

Cells were lysed in Tris‐Buffered Saline (TBS, pH 7.6) (Merck) supplemented with 2% SDS (Carl Roth), 1x Protease Inhibitor Cocktail (Roche) and 1x PhosSTOP Phosphatase Inhibitor Cocktail (Roche). SDS‐PAGE was performed using 20 μg protein per sample on self‐cast tris‐glycine gels followed by the transfer onto a nitrocellulose membrane. Afterwards, the membrane was blocked with PBS + 10% skim milk for 1 h at room temperature. Primary antibody solutions were prepared in PBS + 1% skim milk and applied overnight at 4°C. Secondary antibodies were diluted in PBS + 0.5% skim milk and applied for 1.5 h at room temperature. After detection of the immunoreactive targets using the WesternBright ECL HRP substrate (Advansta), Fiji (version 1.53) was employed for densitometric analysis. The target band was normalized to the total actin detected in the same lane on the respective membrane. Primary antibodies incorporated in this study targeted human p21 Waf1/Cip1 (Cell Signaling Technology, clone 12D1, #2947, 1:1500 dilution), human p53 (Cell Signaling Technology, clone 7F5, #2527, 1:1500 dilution), and actin (MP Biomedicals, clone c4, #SKU0869100‐CF, 1:10000 dilution). Secondary antibodies comprised goat‐anti rabbit horseradish peroxidase conjugates (Promega, #W401B, 1:3000 dilution) and goat anti‐mouse horseradish peroxidase conjugates (Agilent Technologies, #P0447, 1:5000 dilution).

### 2.6 Immunostaining

The following antibodies were used for immunostaining: rabbit anti‐Ki67 (1:200, Thermo Fisher Scientific, # MA5‐14520), rabbit anti‐Plexin‐A1 (1:100, Thermo Fisher Scientific, #MA5‐32804), rabbit anti‐Plexin‐A3 (1:100, Thermo Fisher Scientific, #APR‐093), rabbit anti‐PTK7 (1:100, Thermo Fisher Scientific, #17799‐1‐AP), rabbit anti‐CYB5R1 (1:50, Thermo Fisher Scientific, #11807‐1‐AP), rat anti‐glial fibrillary acidic protein (GFAP) (1:200, Thermo Fisher Scientific, #13–0300), mouse anti‐GFAP (1:200, Merck Millipore, #MAB3402), guinea pig anti‐ionized calcium binding adaptor molecule 1 (IBA1) (1:500, HistoSure, #234308), Alexa Fluor‐conjugated secondary antibodies, including goat anti‐rabbit IgG Alexa Fluor 594 (1:1000, #A‐11012), donkey anti‐rabbit IgG Alexa Fluor 488 (1:1000, #A‐21206), goat anti‐rat IgG Alexa Fluor 488 (1:1000, #A‐11006), donkey anti‐mouse IgG Alexa Fluor 488 (1:1000, #A‐21202), goat anti‐guinea pig IgG Alexa Fluor 633 (1:1000, #A‐21105) were obtained from Thermo Fisher Scientific.

Cells were fixed in 4% PFA for 10 min and subsequently rinsed three times with ice‐cold PBS. Following blocking for 30 min at room temperature using 3% Bovine Serum Albumin (BSA, Carl Roth) in PBS, cells were incubated with primary antibodies diluted in blocking solution at 4°C overnight. Cells were then washed three times in PBS and incubated with Alexa Fluor‐tagged secondary antibodies in 1% BSA in PBS for 1 h at room temperature in the dark. After three washes in PBS, cells were mounted on microscope slides using VECTASHIELD Antifade Mounting Medium containing 4′,6‐diamidino‐2‐phenylindole (DAPI) (Bio‐Techne, # H‐1200‐NB). Coronal sections of mouse brains were prepared at 40 μm thickness using a vibratome (Leica VT1000S, Leica Microsystem) and stored in PBS with 0.05% NaN3 at 4°C until use. Mouse testes were cryosectioned in sagittal planes at 20 μm using a cryostat (Cryostar NX70, Thermo Fisher Scientific) and stored at −20°C until use. The sections were permeabilized with 0.5% Triton X‐100 in PBS (PBST) three times for 5 min each and blocked in a blocking buffer (3% BSA in PBST) at room temperature for 1 h, followed by incubation with primary antibodies in the blocking buffer at 4°C overnight. On the following day, sections were washed in PBST three time for 5 min each, then incubated with Alexa Fluor‐tagged secondary antibodies in the blocking buffer for 2 h in the dark. After three 5‐min washes in PBS, the samples were mounted using VECTASHIELD Antifade Mounting Medium containing DAPI. Imaging was performed on an LSM800 confocal microscope (Zeiss) with 5×, 10×, 20×, and 40× oil immersion lenses. Settings for gain and laser intensity were optimized for representative images and standardized between samples for quantification. Images were processed and analyzed using Imaris v.9.9.1, Fiji, and Zeiss ZEN software.

### 2.7 SA‐β‐gal staining

The SA‐β‐gal activity was determined using the Senescence β‐Galactosidase Staining Kit (Cell Signaling Technology, #9860). For each cell condition and replicate, 1 × 10^5^ cells were seeded into a 24‐well plate. After 24 h, the SA‐β‐gal staining was performed in accordance with the manufacturer's protocol. Cells were then co‐stained with DAPI (Merck) and checked under an Epi‐Scope1‐Apotome fluorescence microscope (Carl Zeiss). Random fields were photographed. The proportion of SA‐β‐gal positive cells (blue cells) was calculated as a percentage of total cells using the Fiji software. For mouse tissue sections, 40 μm brain sections obtained via vibratome and 20 μm testis sections obtained via cryostat, as described in the “Immunostaining” protocol, were used. Following overnight SA‐β‐gal staining at 37°C, tissue samples were co‐stained with DAPI and covered with a coverslip. Staining was examined with an Epi‐Scope1‐Apotome fluorescence microscope. For brain analysis, images from four regions (cortex, dentate gyrus (DG), Cornu Ammonis 1 (CA1), and Cornu Ammonis 3 (CA3)) were acquired to determine the SA‐β‐gal‐positive area. For testis analysis, randomly selected regions were imaged. The percentage of SA‐β‐gal‐positive area relative to the total DAPI‐positive area was calculated using Fiji software.

### 2.8 Cell surface protein extraction

Surface proteins for all cell conditions were extracted using the biotinylation method according to the manufacturer's protocol (Thermo Fisher Scientific, #21331). Briefly, ~2 × 10^7^ cells were washed three times with ice‐cold DPBS supplemented with 1 mM CaCl_2_ and 0.5 mM MgCl_2_ (pH 8), then incubated with 0.5 mg/mL Sulfo‐NHS‐SS‐Biotin (Thermo Fisher Scientific, #21331) for 30 min at 4°C. After biotin labeling, cells were rinsed three times with ice‐cold DPBS (pH 8), then incubated with 0.1 M glycine (Carl Roth) for 30 min at 4°C to quench the remaining unreacted NHS ester. The cells were washed another three times with DPBS (pH 8) and lysed in a lysis buffer comprising 50 mM HEPES (pH 7.4), 150 mM NaCl, 1 mM EDTA, and 1.5% SDS. Lysates were incubated with streptavidin magnetic beads (Thermo Fisher Scientific, #65601) overnight at 4°C with gentle shaking. After extensive washing four times with lysis buffer, the streptavidin‐bound proteins were eluted by boiling in a buffer containing 2% SDS, 100 mM Tris–HCl (pH 7.5), and 0.1 M DTT for 5 min.

### 2.9 Sample preparation for MS analysis

Isolated cell surface proteins as described above were reduced and alkylated prior to processing by a previously described modified protocol for Filter‐Aided‐Sample‐Preparation (FASP) (Scifo et al., 2015) to generate tryptic peptides for subsequent label‐free quantitative MS analysis. Samples were digested overnight with trypsin (1:20; in 50 mM ammonium bicarbonate) directly on the filter columns at 37°C and precipitated using an equal volume of 2 M KCl for depletion of residual detergents. Tryptic peptides were then cleaned, desalted on C18 stage tips, and re‐suspended in 20 μL 1% formic acid (FA), prior to Liquid Chromatography–tandem Mass Spectrometry (LC–MS/MS) analysis. MS runs were performed with three technical replicates.

### 2.10 LC–MS/MS analysis

Tryptic peptides were analyzed on a Dionex Ultimate 3000 Rapid Separation Liquid Chromatography (RSLC) nanosystem coupled to an Orbitrap Exploris 480 MS (Thermo Fisher Scientific). Peptides were injected at starting conditions of 95% eluent A (0.1% FA in water) and 5% eluent B (0.1% FA in 80% acenonitrile (ACN)), with a flow rate of 300 nL/min. They were loaded onto a trap column cartridge (Acclaim PepMap C18, 100 Å, 5 mm × 300 μm i.d., #160454, Thermo Fisher Scientific) and separated by reversed‐phase chromatography on an Acclaim PepMap C18, 100 Å, 75 μm × 25 cm (both columns from Thermo Fisher Scientific) using a 75 min linear increasing gradient from 5% to 31% of eluent B followed by a 20 min linear increase to 50% eluent B. The mass spectrometer was operated in data‐dependent and positive ion mode with MS1 spectra recorded at a resolution of 120 K, mass scan range of 375–1550, automatic gain control (AGC) target value of 300% (3 × 10^6^) ions, maxIT of 25 ms, charge state of 2–7, dynamic exclusion of 60 s with exclusion after 1 time and a mass tolerance of 10 ppm. Precursor ions for MS/MS were selected using a top‐speed method with a cycle time of 2 s. A decision tree was used to acquire MS2 spectra with a minimum precursor signal intensity threshold of 3 × 10^5^ for scan priority one and an intensity range of 1 × 10^4^–3 × 10^5^ for scan priority two. Data‐dependent MS2 scan settings were as follows: isolation window of 2 m/z, normalized collision energy (NCE) of 30% High‐energy Collision Dissociation (HCD), 7.5 K and 15 K resolution, AGC target value of 100% (1 × 10^5^), maxIT set to 20 and 50 ms, for scan priority one and two, respectively. Full MS data were acquired in the profile mode with fragment spectra recorded in the centroid mode.

### 2.11 Database searching

Raw data files were processed with Proteome Discoverer™ software (v2.5.0.400, Thermo Fisher Scientific) using SEQUEST® HT search engine against the Swiss‐Prot® Mus musculus database (v2021‐06‐20) and Homo sapiens (v2021‐06‐20). Peptides were identified by specifying trypsin as the protease, with up to 2 missed cleavage sites allowed and restricting peptide length between 7 and 30 amino acids. Precursor mass tolerance was set to 10 ppm and fragment mass tolerance to 0.02 Da MS2. Static modifications were set as carbamidomethylated cysteine, while dynamic modifications included methionine and N‐terminal loss of methionine, for all searches. Peptide and protein false discovery rates (FDR) were set to 1% by the peptide and protein validator nodes in the Consensus workflow. Default settings of individual nodes were used if not otherwise specified. In the Spectrum Selector node, the Lowest Charge State = 2 and Highest Charge State = 6 were used. The INFERYS rescoring node was set to automatic mode and the resulting peptide hits were filtered for a maximum of 1% FDR using the Percolator algorithm in the Processing workflow. A second‐stage search was applied to identify semi‐tryptic peptides. Both unique and razor peptides were selected for protein quantification. Proteins affected by site, reverse, or potential contaminants were filtered out prior to analysis.

### 2.12 Statistics and data analysis

Statistical analyses of qPCR, Western blot, and staining were performed using GraphPad Prism version 9 (GraphPad Software). Data were presented as mean ± SEM. Statistical differences between two groups were analyzed using unpaired two‐tailed Student's *t* test, and statistical differences among three or more groups were calculated by one‐way analysis of variance (ANOVA) followed by Tukey's post hoc test. Differences were defined statistically significant at *p* < 0.05. Figures were prepared using Illustrator v 26.0 (Adobe) and Biorender.com.

Peptide spectral matches (PSMs) of all identified surface proteins across the various cell conditions and cell surface differentially expressed proteins (DEPs) obtained from at least one senescence condition were used for Principal component analysis (PCA). To address missing values in the technical replicates within the same cell conditions, PSM values were imputed using sklearn's iterative imputer prior to subsequent analysis. The normalized z‐scores, adjusted relative to the non‐senescent control group, of PSMs associated with these identified surface proteins/surface DEPs were used for Pearson's correlation analysis. Clustering analyses were conducted using scaled z‐scores of PSMs, with the scale determined by the logarithm base 10 (log10). To address zero values during the log10 transformation, a constant value of 1 was added to all z‐score values prior to the transformation. In the case of negative values corresponding to downregulated proteins, the log10 of the absolute values was computed and multiplied with −1. PCA was performed using the package “tidyverse” in base R version 4.3.1 and visualized with the “ggplot2” package. Pearson's correlation was analyzed using the R package “corrplot” and visualized by a web‐based application “Chiplot” available at https://www.chiplot.online/. Data for clustering analysis were clustered with the Mfuzz method using the “ClusterGVis” package in R and visualized with the “ggplot2” package. Volcano plots were generated using the package “ggrepal” in R and visualized with the “ggplot2” package. Cellular Component enrichment analyses were performed using Ingenuity Pathway Analysis (Ingenuity Systems) and visualized in “Chiplot” web. Molecular Function and Biological Process enrichment analyses were carried out by Gene Ontology (GO) from the Metascape Database (https://metascape.org/gp/index.html) and visualized by Chord diagrams using the “circlize” package in R or using Cytoscape (https://cytoscape.org/), version 3.9.1. The statistical cutoff for enriched pathways was *p* < 0.05 or Benjamini‐Hochberg adjusted *p* < 0.05 with minimal enrichment at 1.5. The heat maps were constructed in “Chiplot” web. Venn diagrams were generated using the “eulerr” package in R or using “InteractiVenn”, a web‐based tool available at https://www.interactivenn.net/. For the analysis of gene expression fractions and log‐odds ratios (LORs) using published single‐cell transcriptome datasets, the read counts for each gene were preprocessed to be binarized, where a value of 1 indicates gene expression and a value of 0 indicates no expression. The proportion of cells expressing a certain gene was calculated as the ratio of gene‐positive cell counts to total cell counts within a given tissue‐cell type in young or old mice. LORs were calculated in R, including a calculation of the corresponding *p*‐values via Fisher's exact test, under the null hypothesis that the odds ratio is 1. The results were visualized using “ggplot2” and “ComplexHeatmap” packages in R.

## 3 RESULTS

### 3.1 MS‐based proteomic analysis of the senescence‐associated cell surfaceome

**FIGURE 1:**
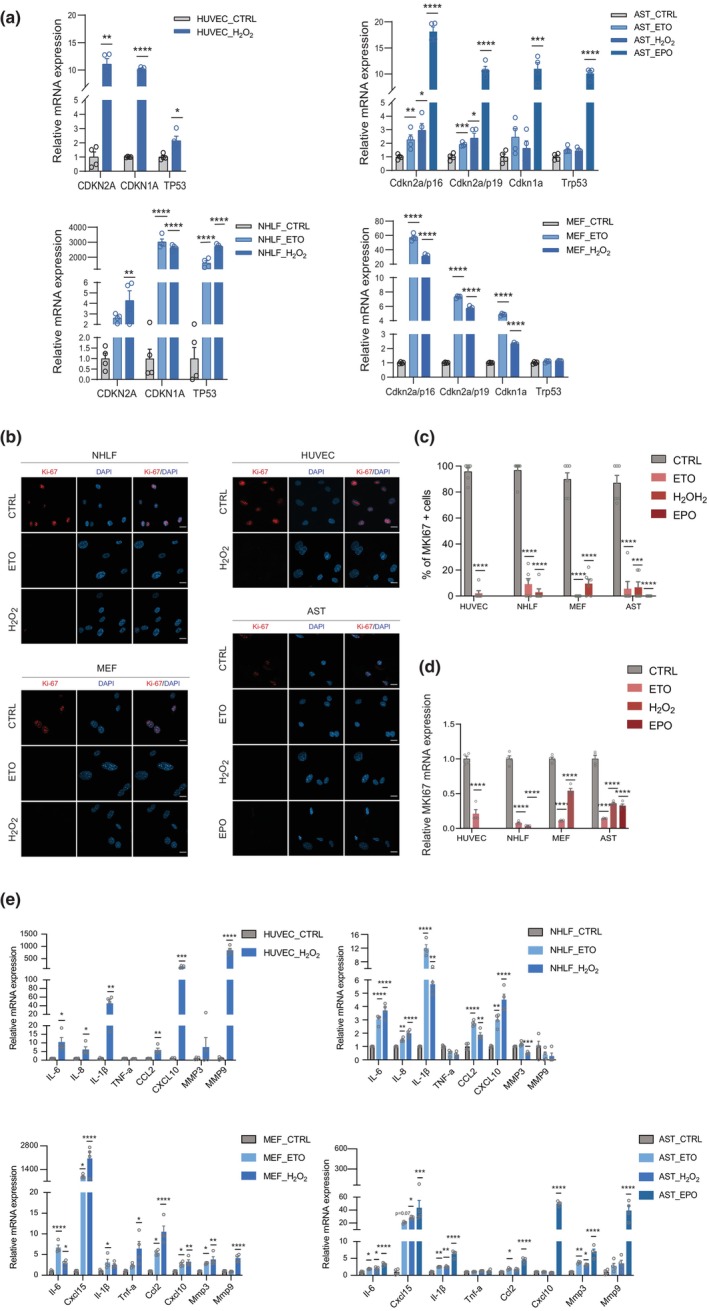
Validation of senescence models. (a,d,e) RT–qPCR analysis of senescence markers, a cell proliferation marker (MKI67), and senescence‐associated secretory phenotype (SASP) markers in DMSO‐treated control cells and senescence‐inducer‐treated cells. The CT value of a given target gene was normalized to the corresponding β‐actin mRNA level with 2^−∆CT^ method for each sample. Data were normalized to the average of the corresponding control group and are presented as the mean ± SEM from four biological replicates. Statistical significance was determined using an unpaired two‐tailed Student's *t* test for comparisons between two groups, and one‐way analysis of variance (ANOVA) followed by Tukey's post hoc test for analyses involving more than two groups, with *p < 0.05; **p < 0.01; ***p < 0.001; ****p < 0.0001. (b) Representative immunofluorescence images of KI67 (red) in control cells and senescence inducer‐treated cells. Cell nuclei were stained with DAPI (blue). Scale bar = 20 μm. (c) Quantitative analysis of KI67‐positive cells, calculated as the ratio of KI67‐positive cells to DAPI‐positive cells per field. Data are presented as the mean ± SEM from six biological replicates. ***p < 0.001; ****p < 0.0001, determined by unpaired two‐tailed Student's *t* test (between two groups) or, in the case of more than two groups, one‐way ANOVA followed by Tukey's post‐hoc test where appropriate. CCL2, chemokine (C‐C motif) ligand 2; CXCL10, chemokine (C‐X‐C motif) ligand 10; Cxcl15, chemokine (C‐X‐C motif) ligand 15; IL‐1β, interleukin 1 beta; IL‐6, interleukin 6; IL‐8, interleukin 8; MKI67, marker of proliferation Ki‐67; MMP3, matrix metallopeptidase 3; MMP9, matrix metallopeptidase 9; TNF‐α, tumor necrosis factor alpha.

We established a proteomic workflow to profile cell surface proteins selectively associated with senescence (Figure S1). Several models of senescence were constructed, including (1) genotoxic stress‐induced senescence in NHLFs, MEFs, and mouse primary astrocytes; (2) oxidative stress‐induced senescence in HUVECs, NHLFs, MEFs, and mouse primary astrocytes; and (3) proteasome stress‐induced senescence in mouse primary astrocytes. These senescence models were then verified using several established CS markers: Senescent cells exhibited elevated positive SA‐β‐gal staining proportions across all models examined (Figure S2). Furthermore, qPCR analysis revealed increased p16, p21, and/or p53 mRNA levels across the different cellular models included in our analysis (Figure 1a). Notably, a dramatic induction of p21 and p53 was detected in the NHLFs treated with ETO and H_2_O_2_, which was associated with very low mRNA levels (CT values exceeding 40) of p21 and p53 in the control cells (Figure 1a). Elevated protein levels of p21 and p53 in the senescent NHLFs were further validated by western blot analysis (Figure S3). We also observed a significant decline in the proportion of cells positive for the cell proliferation marker MKI67, along with reduced mRNA expression of MKI67 in all senescent models, with particularly pronounced changes in H_2_O_2_‐treated human cells and ETO‐treated MEFs (Figure 1b–d). Furthermore, several SASP‐related genes, including interleukin 6 (IL‐6), interleukin 8 (IL‐8, in human cells), chemokine (C‐X‐C motif) ligand 15 (Cxcl15, in mouse cells), interleukin 1 beta (IL‐1β), and chemokine (C‐C motif) ligand 2 (CCL2), exhibited significantly increased mRNA expression under most senescence conditions. Of note, a dramatic and extensive activation of SASPs was observed in H_2_O_2_‐treated MEFs and HUVECs, and EPO‐treated AST. Interestingly, the senescent NHLFs induced by H_2_O_2_ showed decreased expression of matrix metallopeptidase 3 (MMP3) (Figure 1e).

To enrich for cell surface‐associated proteins, we utilized biotin labeling of cell surface proteins. Label‐free quantitative MS analysis was then employed to determine changes in protein expression levels. Cell surface proteins were defined based on their GO annotations for the plasma membrane and/or extracellular regions, as denoted by the web‐based Protein Center platform embedded within the Proteome discoverer software.

**FIGURE 2:**
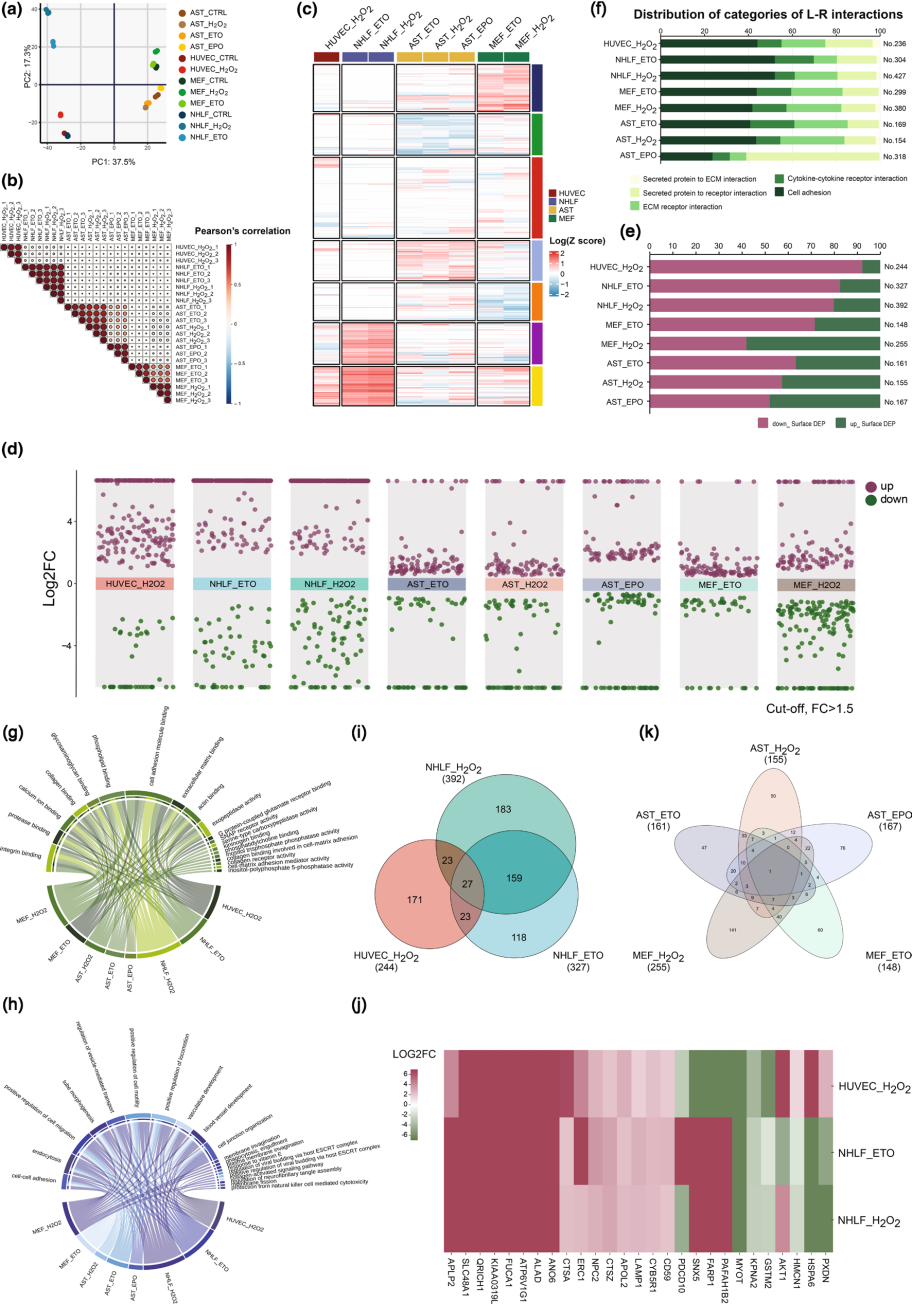
Quantitative analyses of senescence‐associated changes in the cell surfaceome. (a) Principal component analysis (PCA) using the peptide spectral matches (PSMs) of all identified surface proteins (2091 proteins) across twelve cell conditions (senescence and non‐senescence). (b) Heat map of Pearson correlation coefficients (PCCs) comparing correlations across cell types and senescence inducers using the normalized (to corresponding non‐senescence control group) z‐scores of PSMs of all 2091 detected surface proteins. (c) Clustering of expression profiles of the 2091 surface proteins under the different senescence conditions. The scale is set based on the logarithmic base 10 (Log10) transformation of z‐scores of PSMs (for details, see “Materials and methods” section). (d) The multiple volcano plots for differentially expressed cell surface proteins derived from eight analyses comparing expression across senescent versus non‐senescent conditions in various cell types and under different senescence induction regimes. Red and green dots represent the DEPs (upregulated proteins in red and downregulated proteins in green) featuring a significant difference (*p* < 0.05) and a fold change (FC) of at least 1.5 when comparing senescence to control. The y‐axis shows the log2 (FC) of SEN/CTRL. (e) The bar chart depicts the total number of surface DEPs, as well as the proportions of upregulated and downregulated surface DEPs in each group. (f) The total number and distribution of categories of ligand–receptor (L–R) interactions generated by surface DEPs across the eight senescence scenarios studied here. The horizontal axis shows the relative proportions of L–R interaction categories among surface DEPs under the different cell type/inducer conditions. (g, h) These chord diagrams summarize the top 10 most commonly (upper left) and uniquely (upper right) enriched Gene Ontology (GO) terms of molecular function (MF) (g) and biological process (BP) (h) in surface DEPs derived from human and mouse senescent cells. (i) The Venn diagram shows the number of surface DEPs and their overlap across the three human senescence comparisons. (j) This heat map shows senescence‐associated expression changes across the 27 shared human surface DEPs, based on log2 (FC) of the respective SEN/CTRL comparison. (k) Venn diagram of the number of surface DEPs overlapping among the five mouse senescence comparisons included in this study. AST, mouse astrocytes; ECM, extracellular matrix; EPO, Epoxomicin; ETO, Etoposide; H_2_O_2_, hydrogen peroxide; HUVEC, human umbilical vein endothelial cell; MEF, mouse embryonic fibroblast; NHLF, normal human lung fibroblast.

Approximately 32% of the total identified proteins, ranging from 29.1% to 34.9% across cell conditions, were categorized as cell surface proteins (Figure S4). A total of 2091 cell surface proteins were identified across all cell conditions. PCA was carried out to determine how all cell conditions cluster based on the PSM values of these 2091 proteins (Figure 2a, Table S2). The separations based on senescent versus non‐senescent cells were observed in both PC1 and PC2, indicating CS extensively alters the cell surface proteome. Notably, different cell type‐originated senescence conditions exhibited largely distinct distributions. Senescent conditions triggered by different inducers within the same cell type also clustered separately, indicating that both the cell type and nature of the CS inducer have effects on the cell surface proteomic profiles of senescence. Furthermore, Pearson correlation coefficient analyses were conducted to compare correlations between cell conditions against one another, revealing that senescence conditions within the same cell type were highly correlated (Figure 2b). Moreover, the surface protein expression trajectories during senescence were demonstrated in an unsupervised clustering analysis (Figure 2c). The expression profiles of surface proteins in senescence conditions originating from different cell types were largely distinct (Figure 2c).

### 3.2 Quantitative analysis of differential surface proteomic profiling in senescent cells

Next, we conducted a quantitative comparison of cell surface proteomic profiles between senescent cells and their non‐induced counterparts, focusing on proteins with significant changes (*p* < 0.05) and a fold change (FC) of ≥1.5 (senescence/control, SEN/CTRL) (Figure 2d). Across distinct senescence models, this analysis yielded 369–1251 DEPs, of which 31.1%–43.2% were cell‐surface‐associated (Table S3). As expected, the majority of the differentially expressed cell surface proteins showed a significant upregulation. An exception was observed in senescent MEFs induced by oxidative stress, where 58% of cell surface DEPs were downregulated. Notably, senescent NHLFs displayed the highest number of surface DEPs regardless of the inducer. In terms of the magnitude of protein level changes, senescent human cells exhibited more robust changes (based on overall LOG2FC) than senescent mouse cells across the various cell types and inducers (Figure 2d,e). This is consistent with the much higher number of identified DEPs and the more pronounced senescence‐associated phenotypic changes observed in senescent human cells compared to senescent mouse cells (Figure 1). Furthermore, the PCA, Pearson correlation coefficient, and protein clustering analysis using PSM values of all cell surface DEPs obtained from at least one senescence condition (981 proteins in total) revealed that the senescence surfaceome is more strongly determined by the cell type of origin than the CS driver (Figure S5a–c, Table S2).

To determine the molecular features of the CS surfaceome, we performed molecular function enrichment analysis of surface DEPs within each senescent cell model. This analysis was carried out by GO from the Metascape Database (https://metascape.org/gp/index.html) using the GO Molecular Functions resource for both *Homo sapiens* and *Mus musculus*. Figure S5d provides a comparison of significantly (*p* < 0.05) enriched molecular functions across senescence models, with a complete listing of clusters and corresponding terms available in Table S4. The CS surfaceome of human and mouse senescence models was enriched with both shared (179 items) and distinct (115 items unique to *Homo sapiens* and 58 items unique to *Mus musculus*) molecular functions (Figure S5d, Table S4). Figure 2g displays the top 10 commonly enriched clusters across eight senescence conditions as well as the top 10 specifically enriched clusters unique to each senescence condition (ranked by their summed *p*‐values) (Table S4). The most abundant molecular function pathway, universally shared across all CS conditions, was the cell adhesion molecule binding pathway. However, certain CS conditions also exhibited unique pathway enrichments. In particular, the molecular function pathways enriched in H_2_O_2_‐induced senescent NHLFs presented a largely distinctive profile (comprising 34 specifically enriched clusters), featuring transporters, notably those associated with ATPase‐coupled ion transmembrane transporter activity (Table S4).

To gain insight into potential interactome dynamics linked to the CS surfaceome, we employed a ligand–receptor (L–R) interaction analysis utilizing literature‐supported interactions derived from single‐cell RNA sequencing (scRNA‐seq) datasets available at Cellinker (Y. Zhang, Liu, et al., 2021). Across the eight senescence conditions, this analysis revealed 154–427 binding partners, including 67–222 cell‐adhesion interactions, 24–95 extracellular matrix (ECM)–receptor interactions, 17–59 cytokine‐cytokine receptor interactions and 22–193 secreted protein‐receptor interactions, and 1–8 secreted protein to ECM interactions (Table S5). We further explored the distribution of interaction types for each senescence scenario, visually shown in Figure 2f. Consistent patterns of L–R interaction distribution were displayed across the various senescent cell models in both species, with cell‐adhesion interaction representing the most common (40%–52%) and secreted protein to ECM interaction representing the least common interaction (below 3.5%). Apart from cell‐adhesion interactions, receptor‐related interactions (cytokine‐cytokine receptor and ECM‐receptor interactions) were also common (28.3%–44.4%), followed by secreted protein‐associated interactions (14.8%–25%). A noteworthy exception was observed in senescent mouse astrocytes induced by EPO, where secreted protein to receptor interactions constituted the majority (60.7%) of all interactions, contributing to 61% of secreted protein‐associated interactions and 15.4% of receptor‐related interactions (Figure 2f).

To further analyze potential biological implications of the CS surfaceome, we conducted biological process (BP) enrichment analysis of the surface DEPs in each senescence condition using the GO Biological Processes resource for both *Homo sapiens* and *Mus musculus* (Figure S5e, Table S6). The Chord diagram reveals the top 10 enriched clusters (ranked by their summed *p*‐values), both shared and unique, across two species (Figure 2h, Table S6). Several pathways exhibited common involvement in the CS surfaceome across the diverse senescent models. Among these pathways, cell–cell adhesion, regulation of cell migration, and vasculature development have been implicated in CS due to their mechanistic relevance (Kuilman et al., 2010; Levi et al., 2020). Pathways such as vesicle‐mediated transportation and endocytosis are known characteristics of the SASP (Coppé et al., 2010). Senescent NHLFs induced by H_2_O_2_ had the highest number (217) of uniquely enriched clusters, notably association with the “cellular response to increased oxygen levels” pathway (Table S6).

### 3.3 Shared surfaceome contributes to senescence signature

Senescent cells share common features of morphological changes, cell‐cycle arrest and SASP signatures (Hernandez‐Segura et al., 2018). To identify senescence‐associated cell surface molecules shared across diverse senescent cell models, we intersected the respective lists of cell surface DEPs. As shown in Figure 2i, a total of 27 proteins were differentially expressed that overlapped among the three human senescent cell paradigms (Table S7), with 16 proteins (59.2%) exhibiting increased protein expression levels. In contrast, only a small proportion (11.1%) of the shared DEPs in human senescent cells showed a significant decrease in protein abundance across all human cell models (Figure 2j). We also intersected the lists of surface DEPs derived from the different mouse senescence models. Notably, only one protein, N(4)‐(beta‐N‐acetylglucosaminyl)‐L‐asparaginase (AGA), exhibited differential expression (upregulation) shared across all five mouse senescent cell models (Figure 2k). The number of overlapping DEPs among senescence AST conditions dramatically decreased from 74 to 18 when including the EPO inducer. Additionally, 8 DEPs were identified as overlapping among senescent mouse cell conditions without the EPO inducer. Therefore, the significantly fewer overlapping DEPs in the five mouse senescent models compared to the three human senescent models may be attributed to the additional EPO inducer used in mouse cells.

### 3.4 CS surfaceome shows heterogeneity across cell types

**FIGURE 3:**
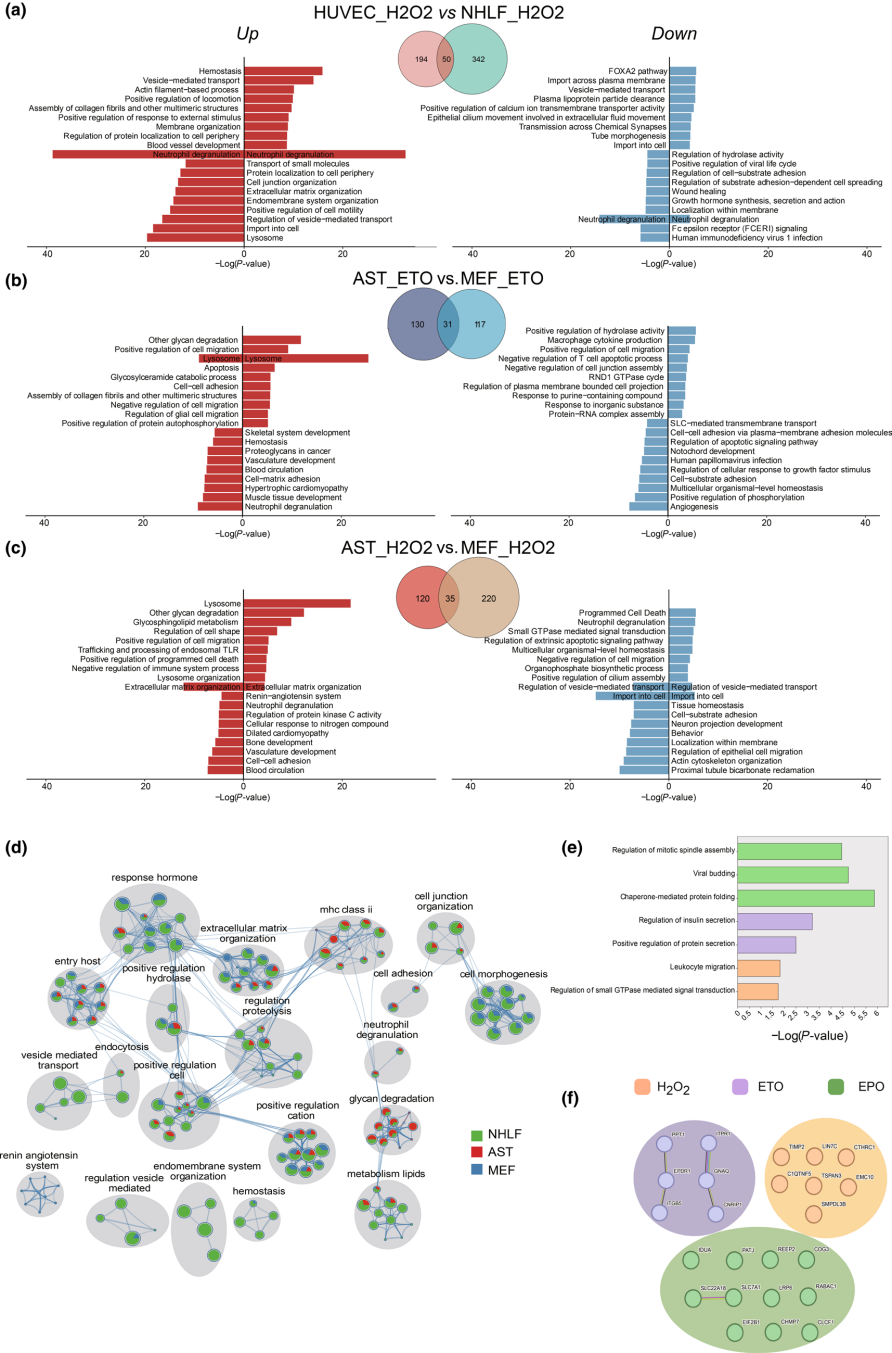
Cell type‐dependent and inducer‐dependent surface protein signatures of senescence. (a‐c) Venn diagrams show the number of differentially expressed surface proteins and their overlap induced by the same senescence induction regime (either oxidative stress or genotoxic stress) across different cell types. Bar charts show the top 10 enriched biological pathways of both upregulated (in red) and downregulated (in blue) cell surface proteins within each senescence comparison. (d) Network plot for the enriched biological pathways of upregulated surface proteins shared by oxidative‐stress‐ and genotoxic‐stress‐induced senescence conditions. The enriched terms with a similarity >0.3 are connected by edges. Each node represents an enriched term shown as pie chart with color coding based on cell type (NHLFs in green, mouse astrocytes in red, and MEFs in blue). The size of a node is proportional to the total number of hits that fall into the term, and the size of a slice in the pie represents the percentage of protein‐coding genes under the term that originated from the corresponding cell type. (e) The chart displays biological pathways enriched among surface proteins uniquely upregulated by specific inducers but shared across cell types, plotted against the level of significance of the enrichment. (f) String protein–protein interaction analysis (https://string‐db.org) of inducer‐specific senescence surface markers shared by different cell types from the present study. Increased proteins induced by replicative exhaustion (reported in published studies) were excluded as these would not be specific to the senescence inducers studied here. The nodes represent surface markers, which are color‐coded according to inducers. The edge represents the type of interactions: Interactions derived from curated databases in blue, co‐expression interactions in black, text‐mining interactions in green, and experimentally determined interactions in pink; the thicker the edge, the higher the confidence.

To determine how different cell types affect the CS surfaceome, we conducted comparative analyses involving surface DEPs in three contexts: (1) senescent human endothelial cells versus lung fibroblasts induced by H_2_O_2_ (Figure 3a); (2) senescent mouse astrocytes versus fibroblasts induced by ETO, and (3) senescent mouse astrocytes versus fibroblasts induced by H_2_O_2_ (Figure 3b,c). Our analysis revealed that only 12.8%–22.6% of DEPs were shared within each comparison, with 37.1%–61.2% of proteins upregulated, 8%–20% downregulated, and intriguingly, 19.4%–42.9% displaying opposing changes after identical CS inducer treatments (Figure 3a–c and Figure S5f–h). To determine whether functional features of the CS surfaceome differ between cell types, we performed biological pathway analysis for both upregulated and downregulated cell surface proteins. The comprehensive and top 10 lists of enriched clusters for each comparison are provided in Table S8. In these comparisons between two distinct cell types subjected to the same senescence inducer, enriched pathways for upregulated proteins exhibited an overlap ranging from 36% to 93%, while for downregulated proteins, the overlap ranged from 25% to 36%. The top 10 ontologies of each condition are visualized in Figure 3a–c, revealing distinct highly‐enriched biological pathways within each comparison. Our analysis highlights heterogeneity in senescence attributed to cell type.

### 3.5 Senescence inducers drive both common and unique CS surfaceome changes

Next, we wanted to compare changes in surface DEPs caused by different senescence inducers in the same cell type. To assess this, we performed comparative analyses of the surface DEP effects induced by the DNA‐damaging reagent ETO versus the Reactive oxygen species (ROS) reagent H_2_O_2_ in senescent human lung fibroblasts, mouse fibroblasts, and mouse astrocytes (Figure S5i). Emphasis was placed on DEPs upregulated under CS. Nearly half of the surface DEPs (ranging from 41.1% to 55.9%) were consistently induced following these two inducers within the same cell type (Figure S5i). Furthermore, the biological process enrichment analysis for the surface DEPs that are shared within each comparison revealed that the robustly activated pathways (based on *p*‐values of enrichment), linked to neutrophil degranulation, cell–cell adhesion, and positive regulation of cation transmembrane transport, are closely associated with both inducers (Figure 3d).

To assess the potential of predicting the originating inducer of CS based on inducer‐specific CS surface markers, we characterized the surface proteomic signatures that were exclusive to different CS inducers (ETO, H_2_O_2_, and EPO), irrespective of cell type (Figure S5i,j). We identified pathways activated across diverse cell types, yet distinctly associated with specific stressors, shown in Figure 3e and Table S9. At the molecular level, we focused on the identification of inducer‐specific CS surface markers exclusively associated with distinct CS drivers and consistently upregulated in at least two distinct cell types (Figure 3f). Given the lack of a RS model in our study, we integrated two previously published datasets derived from RS models. These two datasets included a whole transcriptome study in MEFs (PD 11 vs. PD 6) (Chen et al., 2018) and a surface proteome study in human primary fibroblasts BJ (PD 28 vs. PD 84) (Mrazkova et al., 2018). After exclusion of molecules overlapping with markers upregulated under RS, we identified six proteins, including inositol 1,4,5‐trisphosphate receptor type 1 (ITPR1) and palmitoyl‐protein thioesterase 1 (PPT1), as an ETO‐specific CS surface signature. In the context of H_2_O_2_‐induced senescence, a distinctive signature emerged, comprised of seven proteins, including sphingomyelin phosphodiesterase acid like 3B (SMPDL3B) and collagen triple helix repeat containing 1 (CTHRC1). Moreover, we identified 11 proteins that exhibited robust and exclusive upregulation following EPO induction (Figure 3f, Table S10).

### 3.6 Identification of potential senotherapeutic targets

**FIGURE 4:**
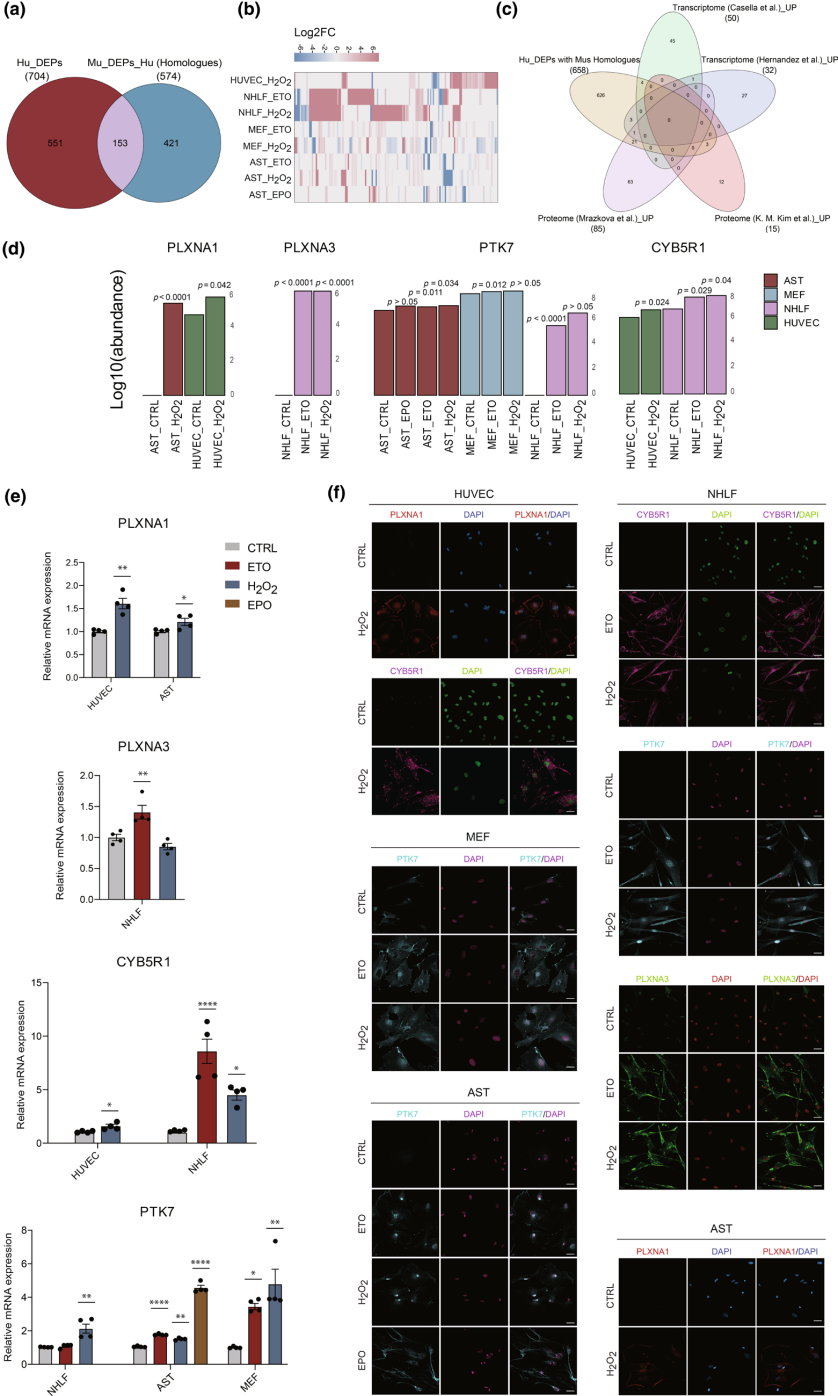
Identification of potential senotherapeutic targets. (a) Venn diagram showing number of surface DEPs present in at least one senescent condition overlapping between *Homo sapiens* and *Mus musculus*. (b) Heat map of the expression profiles of 153 overlapped surface DEPs between two species based on log2 fold change (FC) of SEN/CTRL. (c) Venn diagram comparing human (Hu) senescent cell condition‐derived surface DEPs with murine (Mu) homologues from current study and upregulated senescence signatures identified in published transcriptome and proteome datasets. (d) The average abundance of four potential senotherapeutic targets detected by MS in different senescence conditions and the corresponding control cells. The y‐axis shows the logarithm base 10 (log10) of the abundance. (e) RT–qPCR analysis of our four targets comparing senescent cells and control cells. The CT value of a given target gene was normalized to the corresponding β‐actin mRNA level for each sample. Data were normalized to the average of the corresponding control group and are presented as the mean ± SEM based on four biological replicates. Statistical significance was determined using an unpaired two‐tailed Student's *t* test for comparisons across two groups, and one‐way ANOVA followed by Tukey's post hoc test for analyses involving more than two groups, with *p < 0.05; **p < 0.01; ****p < 0.0001. (f) Expression and cellular localization of our four potential senotherapeutic targets in control and senescent cells were analyzed using immunofluorescence staining. Cell nuclei were stained with DAPI. Scale bar = 40 μm.

Given that ideal senotherapeutic targets should be highly and selectively expressed in senescent cells but not in normal tissues, we explored the expression profiles of shared upregulated CS surface markers in our study, totaling 16 in human cells and 1 in mouse cells, across various human vital tissues, using data provided by the Human Proteome Map (HPM) (Figure S6a) (M.‐S. Kim et al., 2014). Among the considered 17 CS surface markers, cytochrome b5 reductase 1 (CYB5R1) was expressed at relatively low levels across various human tissues, which is consistent with the low CYB5R1 mRNA expression levels observed across adult mouse tissues based on RNA‐seq data from the “European Molecular Biology Laboratory (EMBL‐EBI) Expression Atlas” (Geiger et al., 2013; Merkin et al., 2012) (Figure S6d,e, Table S11). To identify potential surface targets emerging during senescence across *Homo sapiens* and *Mus musculus*, we selected all surface DEPs obtained in at least one senescence condition within each species and determined the corresponding homologues in the other species via the biological DataBase network bioDBnet (tool db2db) (https://biodbnet‐abcc.ncifcrf.gov/db/db2db.php). In total, we identified 704 cell surface DEPs in human senescent cells, 658 of which have murine homologues. Amongst the 589 cell surface DEPs derived from mouse senescent cells, 574 proteins have human homologues. After comparison of both DEP datasets from the two species, a total of 153 overlapping cell surface proteins were identified and comprised of 69 proteins that were upregulated in at least two senescence conditions (Figure 4a,b, Table S12). Furthermore, the expression profiles of these upregulated 69 proteins in adult human and mouse vital tissues were established, with glutathione peroxidase 7 (GPX7), fragile X messenger ribonucleoprotein 1 (FMR1), plexin A1 (PLXNA1), and protein tyrosine kinase 7 (PTK7) demonstrating either absent or low expression levels in both species (Figure S6b,d,e, Table S11). To broaden the search for potential targetable CS surface markers, we compared our human senescent‐cell‐derived cell surface DEPs with murine homologues (in total 658 proteins) to established CS signatures, including two‐transcriptomic (Casella et al., 2019; Hernandez‐Segura et al., 2017) and proteomic (K. M. Kim et al., 2017; Mrazkova et al., 2018) CS datasets. Thirty‐two cell surface molecules were identified that overlapped between our DEPs and at least one of the reported upregulated CS signatures, among which plexin A3 (PLXNA3) expressed at very low levels in various tissues in both human and mice (Figure 4c, Figure S6c–e and Table S11). Consistent with our findings, PTK7 was also identified as a CS surface marker in a RS human fibroblasts BJ model (Mrazkova et al., 2018). Other previously published senescence markers, including the well‐established DPP4 (K. M. Kim et al., 2017) and glycoprotein nonmetastatic melanoma protein B (GPNMB) (Suda et al., 2021), which have been proposed as potential immune‐based senotherapeutic targets, were also detected as DEPs in several senescence conditions in our study.

In summary, we identified four senescence markers from the aforementioned three sources, which are highly expressed on the membrane of senescent cells but are absent or expressed at low levels in normal tissues of both mice and humans. The abundance of these potential senotherapeutic targets detected by MS in different senescence conditions and the corresponding control cells is shown in Figure 4d. The increased mRNA levels of these four markers in senescent models established in our study were validated by qPCR (Figure 4e, Table S13). Furthermore, we determined the protein expression and localization of these targets in senescent models by immunofluorescence staining. As shown in Figure 4f, based on immunofluorescence analyses, PLXNA1 was undetectable in control cells but significantly increased on the surface of H_2_O_2_‐induced senescent HUVECs and AST. PLXNA3 exhibited very low basal expression in proliferative NHLFs but showed marked upregulation in senescent NHLFs. Cell surface PTK7 protein expression appeared to be increased in senescent MEFs, AST, and NHLFs, regardless of the inducer, while being absent or expressed at very low levels in control cells. CYB5R1 was virtually undetectable in proliferative HUVECs and NHLFs, while significantly increased in their senescent counterparts.

### 3.7 Validation of potential senotherapeutic targets in aging and age‐related disease in vivo

**FIGURE 5:**
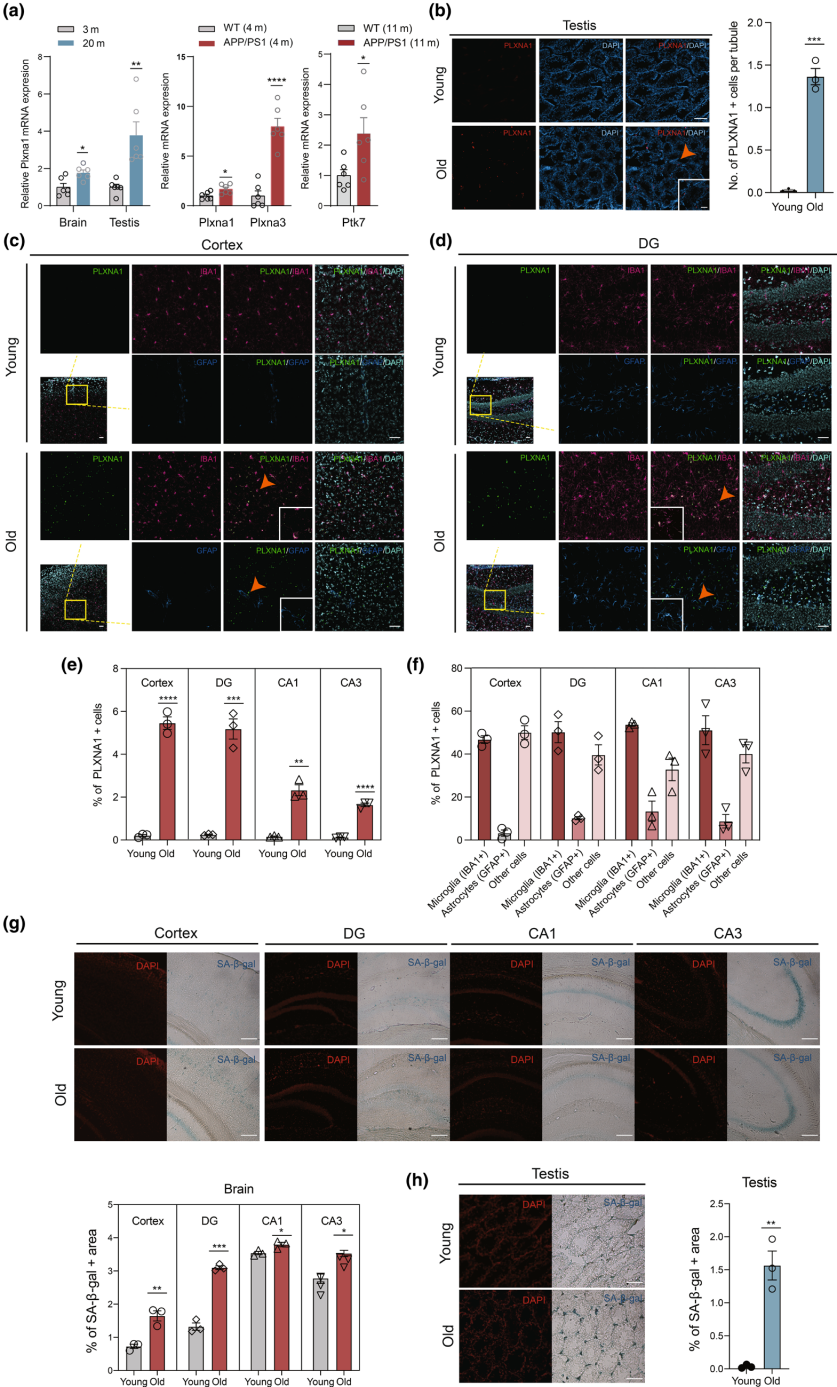
Expression of our senotherapeutic targets in mouse tissues in vivo. (a) RT–qPCR‐based assessment of four potential senotherapeutic targets in tissues derived from young (3‐month‐old) and old (20‐month‐old) wild‐type mice, as well as in brain of APP/PS1 mutant mice. Data are presented as the mean ± SEM based on six biological replicates; normalized to β‐actin mRNA level. *p < 0.05; **p < 0.01; ****p < 0.0001 (unpaired two‐tailed Student's *t* test). (b) Representative immunofluorescence images of PLXNA1 (red) in testes in young and old mice (left panel). Cell nuclei were stained with DAPI (blue). Scale bar = 200 μm. Orange arrow points to an example of PLXNA1‐positive cells in the testicular interstitial space, shown in the high magnification view. Right panel showing quantitative analysis of PLXNA1‐positive cells per seminiferous tubule in young or old mice. Data are based on four to six sections and 30–40 seminiferous tubules per section for each mouse (*n* = 3 mice per age group) and are presented as mean ± SEM. ***p < 0.001 (unpaired two‐tailed Student's *t* test). (c,d) Representative images of triple immunofluorescence staining for PLXNA1 (green), IBA1 (rose red), and GFAP (blue) in the cortex (c) and DG region (d) of young and old mice. Cell nuclei were stained with DAPI. Scale bar = 40 μm. Orange arrows highlight examples of co‐localization of PLXNA1 with IBA1 or GFAP, shown in high magnification views. (e) Quantitative analysis of % PLXNA1‐positive cells in cortex and different regions of hippocampus in young and old mice using 10x objective view fields based on four sections per mouse (*n* = 3 mice per age group). Data are presented as mean ± SEM. **p < 0.01; ***p < 0.001; ****p < 0.0001 (unpaired two‐tailed Student's *t* test). (f) Quantitation of the ratio of PLXNA1‐positive cells co‐expressing IBA1 or GFAP to total PLXNA1‐positive cells in cortex and different regions of hippocampus in old mice, using randomly selected 20x objective view fields. (g, h) Representative images and quantification of SA‐β‐gal staining in different brain regions (g) and testis (h) of young and old mice. Cell nuclei were stained with DAPI. Scale bar = 200 μm. The percentage of SA‐β‐gal‐positive area is relative to the total DAPI‐positive area. *n* = 3 per age group. Data are presented as mean ± SEM. *p < 0.05; **p < 0.01; ***p < 0.001 (unpaired two‐tailed Student's *t* test). CA1, Cornu Ammonis 1; CA3, Cornu Ammonis 3; DG, dentate gyrus.

CS has been described as a mechanism contributing to aging and multiple age‐related pathologies (He & Sharpless, 2017). To assess the expression of our candidates in whole aging tissues in vivo, we measured the mRNA expression levels of four of our potential candidates, PLXNA1, PLXNA3, PTK7, and CYB5R1, which are expressed on the cell membrane with extracellular epitopes (schematic visualization of six candidates is available through Protter: https://wlab.ethz.ch/protter/start/ and shown in Figure S7), in young and old wild type (WT) mice, as well as in brains of APP/PS1 mutant mice. As shown in Figure 5a, qPCR analysis revealed significantly elevated PLXNA1 mRNA in brain and testis of old mice (20‐month‐old) compared to young mice (3‐month‐old). Although not statistically significant, PTK7 and PLXNA3 exhibited trends of upregulation by at least 1.5‐fold in brain (Table S14). Additionally, our MS‐based proteomic analysis (unpublished data) confirmed increased expression levels of PLXNA1 protein in the kidneys of aged male WT mice (26‐month‐old) relative to young adult mice (3‐month‐old) (Table S15). Furthermore, in comparison to WT mice, increased expression of PLXNA1 and PLXNA3 were observed in the AD mouse brain at 4 months of age, while PTK7 expression was elevated at 11 months of age (Figure 5a). To validate the protein expression of PLXNA1 in brain and testis in old WT mice, we performed immunofluorescence staining to analyze the proportion and location of PLXNA1‐positive cells in 20‐month‐old mice compared to 3‐month‐old mice. PLXNA1 was virtually undetectable in the brains of young mice, whereas old mice exhibited PLXNA1‐positive cells ranging from 1.64% to 5.45% across the cortex and different regions of hippocampus (Figure 5c–e, Figure S8). Notably, PLXNA1‐expressing cells were preferentially located in the cortex and DG region in old mice (Figure 5e). This distribution is consistent with the increased presence of SA‐β‐gal‐positive cells observed in these regions (Figure 5g), indicative of senescence. Furthermore, co‐staining with glial markers revealed that over 45% and 50% of PLXNA1‐positive cells in the cortex and hippocampus, respectively, were microglia in old mice (Figure 5f). In the testes, PLXNA1‐positive cells were rare in young mice but increased in old mice (Figure 5b). Interestingly, almost all PLXNA1‐expressing cells in old testes were located in the interstitial space outside of seminiferous tubules, in line with the distribution of SA‐β‐gal‐positive cells in these regions (Figure 5b,h).

To further investigate the relative abundance of cells expressing one of the four identified potential senotherapeutic targets in vivo, we analyzed the abundance of cells positive for these candidates as well as CDKN2A (p16ink4a)‐positive cells across different tissue‐cell types in young and old wild‐type mice. We utilized the droplet single‐cell transcriptomic dataset Tabula Muris Senis (TMS) (Tabula Muris Consortium, 2020) which includes over 245,000 annotated cells from 16 tissues of male and female mice from six age groups. Young mice were defined as ≤3‐month‐old and old mice as ≥18‐month‐old. Tissues represented only in young or old age groups (skin of body, pancreas, large intestine, fat, and trachea) were excluded, resulting in 130 cell types from 11 tissues for analysis. For brain cells, we also included an established droplet transcriptomic dataset for the mouse brain (Ximerakis et al., 2019), encompassing both young (2–3‐month‐old) and old (21–22‐month‐old) male mice with 25 annotated cell types. Merging these two datasets resulted in 155 tissue‐cell types from 12 tissues included in these analyses.

**FIGURE 6:**
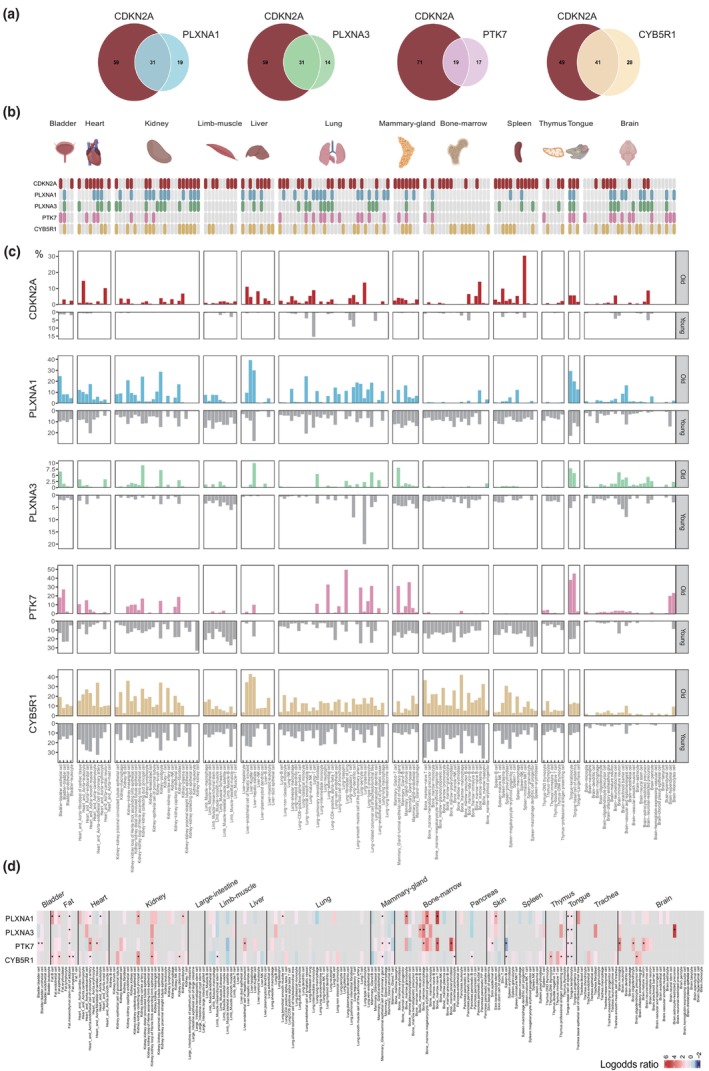
Age‐dependent increase in the abundance of cells positive for our senotherapeutic targets and/or CDKN2A in various tissue‐cell types in vivo. (a) Venn diagrams showing the number of tissue‐cell types with an age‐dependent increase in the proportion of CDKN2A‐positive cells and their overlap with those displaying an age‐dependent increase in the proportion of senotherapeutic target‐positive cells in old mice compared to young mice. (b) Comparison of tissue‐cell types with an age‐dependent increase in CDKN2A‐positive cells (red) to those with an age‐dependent increase in senotherapeutic target‐positive cells (blue for PLXNA1, green for PLXNA3, pink for PTK7, yellow for CYB5R1) across various tissue‐cell types. (c) Bar charts showing the proportions of cells expressing each gene within a given tissue‐cell type in young (gray) and old (blue for PLXNA1, green for PLXNA3, pink for PTK7, yellow for CYB5R1) mice. % indicates the proportion of cells expressing each gene within the total cell count for a given tissue‐cell type. (d) Heat map of log‐odds ratios (LORs) determining the correlation between the presence of the CDKN2A gene and each candidate within a given tissue‐cell type. A positive LOR indicates a positive correlation, while a negative LOR indicates a negative correlation. Statistical significance was determined by Fisher's exact test, with *p < 0.05.

As shown in Figure 6a–c, the proportion of cells expressing CDKN2A increased in 90 out of 155 tissue‐cell types (58%) in old mice compared to young mice. Notably, spleen immature NKT cells (with the highest proportion of 30.36%), heart leukocytes, liver endothelial cells, lung and bone marrow plasma cells, and tongue keratinocytes showed a significant increase in CDKN2A‐positive cells. Fifty cell types (32.3%) exhibited an age‐dependent increase in the proportion of PLXNA1‐positive cells, with dramatic increases observed in lung dendritic cells, club cells of bronchioles, lung plasma cells, and brain pericytes in old mice, while being absent in young mice. Among these 50 tissue‐cell types, 31 (62%) overlapped with those showing an age‐dependent increase in CDKN2A‐positive cells. Furthermore, 45 cell types (29%) displayed an age‐dependent increase in PLXNA3‐positive cells, with increased abundance in kidney collecting duct principal cells, hepatic stellate cells, and astrocyte‐restricted precursors, while being absent in young mice. Of these, 31 cell types (68.9%) overlapped with those showing an age‐dependent increase in CDKN2A‐positive cells. Additionally, 36 cell types (23.2%) showed an age‐dependent increase in PTK7‐positive cells, with notably higher abundance (>30%) in lung adventitial cells, lung fibroblasts, and mammary gland basal cells in old mice, with 19 cell types (52.8%) overlapping with those showing an age‐dependent increase in CDKN2A‐positive cells. Strikingly, 69 cell types (44.5%) exhibited an age‐dependent increase in CYB5R1‐positive cells, with dramatic increases observed in kidney plasma cells, club cells of bronchioles, and olfactory ensheathing glia, while being absent in young mice. Among these, 41 cell types (59.4%) overlapped with those showing an age‐dependent increase in CDKN2A‐positive cells. Similar to CDKN2A, PLXNA3‐positive cells showed very low proportions across all tissue‐cell types in young mice. Notably, four tissue‐cell types, including kidney collecting duct principal cells, mammary gland luminal epithelial cells, marrow erythroid progenitors, and tongue basal cells of the epidermis, exhibited an age‐associated increase in the proportion of CDKN2A, and all four candidate‐positive cells. Cell counts and cell proportion metrics for each gene are shown in Table S16.

To further address how our four potential senotherapeutic targets overlap with CDKN2A expression in vivo, we calculated log odds ratios (LOR) based on the binarized single‐cell gene expression data for each cell type (see Methods). All the tissue‐cell types (in total 194 cell types) were included in this analysis. A positive LOR indicates a positive correlation, while a negative LOR indicates a negative correlation. Across various tissue‐cell types, we observed both positive and negative correlations between the presence of CDKN2A and our candidates (Figure 6d). Notably, nearly all significant correlations were positive, with the exception of a negative correlation of PTK7 and CDKN2A presence in Spleen B cells. All four candidates displayed significant positive correlations with CDKN2A in adipose mesenchymal stem cells, basal cells of the epidermis and keratinocytes of the tongue. Remarkably high and positive correlations were observed in bone marrow cells, particularly for PLXNA1 and PTK7. PLXNA3 showed predominantly positive correlations with CDKN2A in astrocyte‐restricted precursors, consistent with the age‐dependent increase of PLXNA3‐ and CDKN2A‐positive cells in this cell type. LOR values and corresponding *p*‐values for each gene across all tissue‐cell types are provided in Table S16.

## 4 DISCUSSION

Cell surface proteins act as vital molecular mediators, facilitating crucial cellular functions such as cell–cell communication and metabolite transport. Notably, 66% of drugs approved in the DrugBank database target cell surface proteins (Bausch‐Fluck et al., 2018). The surfaceome is highly context‐dependent and varies extensively across distinct pathological conditions, reflecting its diverse and dynamic functions (Bausch‐Fluck et al., 2018). Senescent cells differ from proliferating cells and display distinct metabolic, as well as protein expression patterns (Hernandez‐Segura et al., 2018). Critical to understanding the mechanisms that shape the senescent phenotype is the CS surfaceome which governs interactions with the extracellular microenvironment.

Our findings indicate that the surfaceome is strongly influenced by CS, revealed by the sizeable expression differences between senescent versus non‐senescent states. The extensive CS‐associated changes in surfaceome expression profiles are in line with alterations in a number of cellular functionalities. Senescent cells, for instance, have altered cell adhesion capabilities, which are closely linked to cytoskeletal reorganization, morphological changes and changes in cell migration rates (Brauer et al., 2023; Levi et al., 2020). Our characterization of the senescent cell surfaceome across diverse conditions revealed, among differentially expressed surface proteins, an abundant enrichment of cell adhesion molecules and demonstrated cell‐adhesion interactions as the most predominant L–R interaction type. The notable prevalence of biological pathways related to cell migration, locomotion, and cell–cell adhesion indicates that cell adhesion and migration are fundamental characteristics of the CS surfaceome. These features significantly contribute to the compositional remodeling of the extracellular matrix. A total of 93 cell surface proteins linked to CS‐related cell adhesion were identified through biological function GO annotation in this study (Table S17). Intriguingly, most of the cell‐adhesion molecules with altered expression in diverse senescence conditions across both species, including ICAM1, serpin family B member 8 (SERPINB8), Thy‐1 membrane glycoprotein (THY1), and Nexilin (NEXN), were found to be upregulated, in line with a role of altered cell adhesion processes in CS. Moreover, the CS surfaceome, positioned at the interface of intracellular and extracellular compartments, exhibited a notable enrichment of the biological pathway “vesicle‐mediated transport,” with 107 proteins identified through GO annotation (Table S17). This finding, together with the involvement of secreted protein‐associated interactions in the L–R interactome, highlights changes associated with the SASP as an additional feature of the CS surfaceome. This is consistent with previous studies demonstrating that some CS surface markers are implicated in the SASP (Hoare et al., 2016; K. M. Kim et al., 2018).

The shared surfaceome may contribute to a more general senescence signature. Within the shared CS surface signature identified in the present study, upregulated proteins, such as V‐type proton ATPase subunit G 1 (ATP6V1G1) and lysosomal protein Lysosome‐associated membrane glycoprotein 1 (LAMP1) have been previously linked to CS (Kang et al., 2017; Rovira et al., 2022). Additionally, our study identified novel markers that were upregulated during CS, including CYB5R1, anoctamin‐6 (ANO6), amyloid beta precursor like protein 2 (APLP2), NPC intracellular cholesterol transporter 2 (NPC2), and transcriptional regulator QRICH1 (QRICH1). Notably, AGA, an enzyme involved in protein metabolism with peptidase and hydrolase activity (Harkke et al., 2003), displayed widespread and significant upregulation across diverse mouse senescent cell models, in line with enhanced lysosomal activity in CS. In the shared CS surface signature that covers downregulated protein targets, glutathione S‐transferase Mu 2 (GSTM2), a detoxification enzyme crucial in metabolizing ROS metabolites and xenobiotics, has been reported to decline during senescence in human primary fibroblasts. This phenotype can be reversed by transfection of small extracellular vesicles (sEVs) carrying recombinant GSTM2 derived from healthy young donors (Fafián‐Labora et al., 2020). The decrease in the expression of GSTM2 across different CS conditions in our study indicates a reduced resistance to oxidative stress in senescent cells.

CS heterogeneity was initially revealed by the varied senolytic susceptibilities among senescent cells (L. Zhang et al., 2023; Zhu et al., 2015). Moreover, it is also exhibited by the diversity in transcriptomic profiles (Casella et al., 2019; Hernandez‐Segura et al., 2017) and SASPs (Basisty et al., 2020), both of which can be attributed to inducers, cell types, and senescence stages. Here, by measuring the CS surfaceome across distinct cell types under a common stimulus, we have demonstrated the profound influence of cell type on shaping the CS surfaceome at both molecular and predicted functional levels of analysis. PCA, Pearson correlation, and clustering analyses consistently indicated that the heterogeneity of the CS surfaceome is preferentially determined by the respective cell type. For instance, the glial cell migration pathway was significantly enriched in senescent astrocytes, notably with brain‐specific proteins like tyrosine phosphatase receptor type z1 (PTPRZ1). Conversely, senescent fetal‐derived fibroblasts exhibited enriched upregulated proteins linked to muscle and skeletal system development, such as matrix metalloproteinase 2 (MMP2) and matrix gla protein (MGP). In the context of oxidative stress‐induced senescent human endothelial cells, notable links to blood vessel development emerged, such as elevated angiogenesis‐related proteins like bone morphogenetic protein 6 (BMP6) and angiopoietin 2 (ANGPT2). This heterogeneity in CS surface profiles among different cell types may arise from differences in cell‐intrinsic responses to senescence‐inducing stimuli. Additionally, in vivo, the contextual interactions of cells within their microenvironment, including neighboring cells and the extracellular matrix, could further modulate the senescence surfaceome in a cell type‐specific manner.

Senescent cells undergo inducer‐dependent changes in autophagy, proteasome activity, and metabolism (Capasso et al., 2015). In this study, we characterized the inducer‐specific CS surfaceome to dissect potential differences induced by various senescence triggers. Notably, DNA damage (ETO)‐driven CS surface profiles were marked by predicted abnormalities in glucose metabolism, while oxidative stress (H_2_O_2_)‐induced CS surfaceome changes were predicted to result in extensive changes in cell signaling. These differential effects may contribute to distinct cellular stress responses and DNA damage repair processes associated with nDNA and mtDNA damage during senescence. Intriguingly, the EPO‐specific CS surfaceome, with its unique L–R interactome pattern in mouse astrocytes, is closely linked to chromosome missegregation, thereby bridging proteasome activity with genomic instability. Furthermore, the inducer‐specific markers identified in this study could be used as biomarkers in the context of chemotherapy‐induced CS in vivo. For instance, ETO and EPO, especially ETO, have been widely studied for cancer treatment (Adams, 2004; Zhang, Gou, et al., 2021). Our inducer‐specific CS surface biomarkers may serve as potential targets to enhance their therapeutic efficacy and minimize side effects, while optimizing administration strategies.

Ideal senotherapy targets should be highly and specifically expressed on senescent cells while sparing normal vital tissues. Among our potential candidates, four cell‐surface proteins with predicted extracellular epitopes exhibiting relatively low or absent expression across diverse vital tissues emerged. PLXNA3, a member of the plexin family, serves as a key cell‐surface receptor for semaphorin signaling and has demonstrated increased transcript levels in previous studies of human senescent cell culture studies (Brauer et al., 2023; Hernandez‐Segura et al., 2017; Schwartz et al., 2021). In vivo, PLXNA3 has been mainly studied in neuronal development and axon guidance, and a link between PLXNA3 and neuronal apoptosis has been demonstrated in PLXNA3 knock‐out mice (Ben‐Zvi et al., 2008). Another plausible candidate that emerged from our study is PTK7, a receptor tyrosine kinase (RTK) family member, which contributes to cell migration and planar cell polarity. Increased protein levels of PTK7 have been observed in RS‐related human fibroblasts (Mrazkova et al., 2018). Moreover, secreted PTK7 was identified as a SASP factor in aging intestines and senescent intestinal fibroblasts (Yun et al., 2023). Intriguingly, all four candidates have been implicated in cancer. Notably, PLXNA1, PLXNA3, and PTK7 are abundantly expressed in multiple cancer types, primarily correlated with aggressive phenotypes and poor prognosis (Jin et al., 2014; Woischke et al., 2016; Zhang, Shao, & Li, 2020). PTK7‐based immunotherapies, such as therapies based on CAR T cells or antibody‐drug conjugates (ADCs), have shown promising preclinical efficacy and tolerability for several solid cancers (Jie et al., 2021; Lee et al., 2023; T. Xu et al., 2023).

Understanding the expression of these candidates in aging in vivo is critical for clinical applications. Our study observed significantly increased PLXNA1 expression and cellular populations expressing PLXNA1 in the brain and testis of old mice compared to young mice, with a distribution similar to that of SA‐β‐gal‐positive cells. Notably, this age‐related increase in PLXNA1‐positive cells was pronounced in microglia in the cortex and hippocampus. Previous studies have revealed a significant increase in nitric oxide production in response to lipopolysaccharide in PLXNA1‐expressing microglia compared to PLXNA1‐deficient microglia, both in vitro (Ito, Yoshida, et al., 2014) and in the hippocampus of mouse brain in vivo (Ito, Morita, et al., 2014). Together with our results, this suggests the potential involvement of PLXNA1 upregulation in microglia senescence‐associated aging pathologies. In the testis, the proportion of SA‐β‐gal‐positive cells located in the interstitial space markedly increased with aging, consistent with previous findings (Ozawa et al., 2023). Histologically, this region contains Leydig cells, endothelial cells, and macrophages, all of which can undergo senescence and have been reported to contribute to age‐related changes in spermatogenic activity and testosterone secretion (Nie et al., 2022; Ozawa et al., 2023; Zhang, Xie, et al., 2020). Future studies are warranted to determine the PLXNA1‐coexpressing cell types in old testes. Furthermore, our in vivo single‐cell transcriptome‐based analysis revealed a substantial overlap between cell types exhibiting age‐dependent increases in CDKN2A‐expressing cells and those expressing our potential senotherapeutic targets, as well as positive correlations between the expression of CDKN2A and our candidates in various tissue‐cell types, suggesting that our results may capture the tissue‐cell‐type‐specific co‐expression patterns of senescence markers and our candidates in vivo. Surprisingly, we did not observe an age‐related increase in PLXNA1‐expressing microglia in this analysis (1.82% vs. 2.11%, Figure 6c). This may be due to the calculation of the fraction of cells expressing PLXNA1, which does not account for the spatial location of the microglia, instead aggregating microglia from the entire brain for analysis. Consistent with previous gene network analyses showing an enrichment of senescence‐associated genes in fibroblasts, endothelial cells, and immune cells in non‐diseased human tissues (P. Xu et al., 2022), our study found that the abundance of cells expressing our four candidate markers preferentially increased in epithelial cells, immune cells, and fibroblasts in aged mice. These observations underscore the potential application of our candidates as senotherapeutic targets to address aging‐related changes and pathologies in vivo.

In summary, our comprehensive investigation of the CS surfaceome highlights CS surface protein changes consistent with pronounced alterations in cell mechanics and compositional ECM remodeling beyond the canonical SASP. The in‐depth characterization of CS surfaceome heterogeneity, shaped by cell type and senescence inducer, lays the foundation for the development of a more rational targeted immune‐based senotherapy. Future endeavors could leverage advanced technologies such as imaging flow cytometry and single‐cell proteomics to further expand our understanding of senescence surface signatures in vivo. The potential utility of the CS surface markers we identified, which exhibit both shared and tissue‐specific upregulation patterns, in targeting senescent cells in aging and age‐related diseases in vivo, will be the focus of forthcoming research.

## AUTHOR CONTRIBUTIONS

Dan Ehninger conceived the research project and provided supervision; Dan Ehninger, Enzo Scifo, Yushuang Deng and Ting Liu planned the research project; Yushuang Deng, Enzo Scifo, Ting Liu, Tao Li, and Kan Xie established assays and performed experiments; Yushuang Deng, Ting Liu, Enzo Scifo, Tao Li, Bernd Taschler, Sarah Morsy, and Kan Xie analyzed data; Dan Ehninger, Yushuang Deng, Enzo Scifo, Ting Liu, Tao Li, Daniele Bano, Kan Xie, Sarah Morsy, and Armin Ehninger contributed interpretation and discussion; Kristina Schaaf and Kan Xie provided technical and scientific support; Yushuang Deng, Enzo Scifo, Kan Xie, Ting Liu, Tao Li and Dan Ehninger drafted the manuscript; Armin Ehninger, Daniele Bano and Dan Ehninger provided resources. All authors agreed on the final version of the paper.

## CONFLICT OF INTEREST STATEMENT

None declared.

## Supporting information


Table S1.

Table S2.

Table S3.

Table S4.

Table S5.

Table S6.

Table S7.

Table S8.

Table S9.

Table S10.

Table S11.

Table S12.

Table S13.

Table S14.

Table S15.

Table S16.

Table S17.

Figure S1.

## Data Availability

Raw MS data is publicly available under the following accession numbers‐ProteomeXchange (PXD048921) and jPOST (Okuda et al., 2017) (JPST002467) via the following link: https://repository.jpostdb.org/entry/JPST002467.0. The R code used in this study for in vivo single‐cell transcriptome‐based analysis is available in the GitHub repository at the following link: https://github.com/ehningerd/Deng_et_al‐senescence_surfaceome. The rest of the data presented here are available from the corresponding authors upon reasonable request.
