# Supplementary material for: Analysis of the senescence‐associated cell surfaceome reveals potential senotherapeutic targets
Source: Aging Cell. 2024 Sep 3;23(12):e14312. doi: 10.1111/acel.14312 (PMC11634743; doi:10.1111/acel.14312)
Supplement: Supplementary file 18 — Figure S1. [file ACEL-23-e14312-s017.pdf]

Supplementary Figure Legends

Figure S1 Schematic of proteomic workflow for identification of senescence associated surface proteins. Senescent cell models were induced in cultured primary human endothelial cells, lung fibroblasts, mouse forebrain astrocytes, and mouse embryonic fibroblasts by either genotoxic stress, oxidative stress, or proteasome stress. Proliferative control cells were DMSO-vehicle treated. Cell surface proteins were extracted from all cell conditions using a biotinylation labeling method, and subsequently subjected to label free mass spectrometric analysis, followed by identification of differentially expressed proteins (DEPs), as well as comprehensive bioinformatics and pathway analyses. Potential senotherapeutic targets were identified from three sources: 1) surface DEPs upregulated across diverse senescence conditions in our study; 2) surface DEPs upregulated in both *Homo sapiens* and *Mus musculus* in our study; 3) surface DEPs upregulated in our study and shared with established CS signatures from previous studies. These identified senescence cell surface markers were prioritized based on their absent or low expression in normal tissues of both mice and humans. The expression and localization of these targets were examined in the senescent cell models established in this study *in vitro* and further analyzed in various tissues of aged wild-type (WT) mice, as well as in the brains of an Alzheimer's disease (AD) mouse model *in vivo*. This figure was created with BioRender (biorender.com). MS, mass spectrometry; YG, young; OD, old.

Figure S2 Senescence associated beta-galactosidase (SA-β-gal) staining of primary cultured cells. (a-d) Representative images of cells treated with DMSO (control) and senescence inducers, co-stained for SA-β-gal activity and DAPI. The SA-β-gal staining was visualized under bright-field microscopy, with blue cells indicating SA-β-gal positivity. DAPI fluorescence staining was employed to aid in the quantification of the percentage of SA-β-gal-positive cells. Scale bar = 100 μm. (e) Quantification of SA-β-gal-positive cells was performed by calculating the ratio of blue cells to DAPI-positive cells in cells treated with senescence inducers compared to those treated with DMSO. Quantitative data are presented as the mean ± SEM from six biological replicates. ****$P < 0.0001$, determined by unpaired two-tailed Student's t-test (between two groups) or, in the case of more than two groups, one-way analysis of variance (ANOVA) followed by Tukey's post-hoc test where appropriate. HUVEC, human umbilical vein endothelial cell; NHLF, normal human lung fibroblast; AST, mouse astrocytes; MEF, mouse embryonic fibroblast; H2O2, hydrogen peroxide; ETO, Etoposide; EPO, Epoxomicin; DAPI, 4′,6-diamidino-2-phenylindole.

Figure S3 Western blot analysis (left panel) and quantification (right panel) of protein expression of P21 and P53 in NHLFs with or without ETO or H2O2 treatment. The band intensity of a given target protein was normalized to the corresponding actin signal for each sample. Data were normalized to the average of the corresponding control group and are presented as the mean ± SEM, from three or four biological replicates. Statistical significance was assessed using one-way ANOVA followed by Tukey's post-hoc tests, with $^{*}P < 0.05$; $^{**}P < 0.01$. NHLF, normal human lung fibroblast; H2O2, hydrogen peroxide; ETO, Etoposide.

Figure S4 Distribution of cellular components of identified proteins in twelve cell conditions. The horizontal axis represents the percentage of identified proteins. HUVEC, human umbilical vein endothelial cell; NHLF, normal human lung fibroblast; AST, mouse astrocytes; MEF, mouse embryonic fibroblast; H2O2, hydrogen peroxide; ETO, Etoposide; EPO, Epoxomicin.

Figure S5 (a) Principal component analysis (PCA) using the peptide spectral matches (PSMs) of all cell surface proteins that were differentially expressed in at least one senescence condition (overlapping among three technical replicates within each cell condition), encompassing a total of 981 proteins. (b) Heat map displaying Pearson correlation coefficients (PCCs) from pairwise correlation analyses of 981 differentially expressed cell surface proteins (DEPs) under various senescence conditions. The analyses are based on normalized z-scores, adjusted relative to the non-senescent control group, of PSMs associated with these DEPs. (c) Clustering of expression profiles for 981 cell surface DEPs across various senescence conditions, with the scale determined by the logarithm base 10 (log10) of the z-scores of PSMs. (d,e) The heat maps show -log(p-values) derived from Gene Ontology (GO)-based molecular function (MF) and biological process (BP) enrichment analyses of surface DEPs plotted against the corresponding MF or BP process. (f,g,h) The heat maps present, for each comparison shown in Figure 3a-c, the senescence-associated expression changes for overlapping surface DEPs based on log2 fold change (FC) of SEN/CTRL. (i,j) Venn diagrams show the number of upregulated surface proteins overlapping between senescence comparisons, in which the effects of different senescence-inducing stimuli (oxidative stress, genotoxic stress, and proteasome stress) are compared within the same cell type. HUVEC, human umbilical vein endothelial cell; NHLF, normal human lung fibroblast; AST, mouse astrocytes; MEF, mouse embryonic fibroblast; H2O2, hydrogen peroxide; ETO, Etoposide; EPO, Epoxomicin.

Figure S6 Heat maps of expression profiles in normal human vital tissues as cataloged by the Human Proteome Map (https://www.humanproteomemap.org/)

of (a) upregulated senescence surface markers shared across all senescence models in human cells (16 proteins) or mouse cells (1 protein) based on the current study; (b) 69 upregulated surface proteins obtained from at least one senescence condition, overlapping between *Homo sapiens* and *Mus musculus* (for details, see Results 3.6); and (c) 32 cell surface molecules common to DEPs derived from senescent human cells (that are differentially expressed in at least one senescence condition), which have murine homologues and also match the upregulated senescence markers from published transcriptome and proteome datasets (for details, see Results 3.6). (d,e) Heat maps showing expression profiles of six identified potential senotherapeutic targets in adult mouse vital tissues based on published RNA-sequence data (d), and in human vital tissues based on the Human Proteome Map combined with the expression profiles of known Chimeric Antigen Receptor T cell (CAR T cell) therapeutic targets in clinical trials (e). The y-axis of the presented data displays the names of the proteins. PLXNA1, plexin A1; PLXNA3, plexin A3; PTK7, protein tyrosine kinase 7; CYB5R1, cytochrome b5 reductase 1; FMR1, fragile X messenger ribonucleoprotein 1; GPX7, glutathione peroxidase 7; ROR1, receptor tyrosine-protein kinase-like orphan receptor 1; ERBB2, receptor tyrosine-protein kinase erbB-2; MSLN, mesothelin; EGRR, epidermal growth factor receptor.

Figure S7 The schematic representation of the protein sequences for six identified potential senotherapeutic targets, each annotated with its respective UniProt accession number, was generated using the Protter protein-visualization platform (https://wlab.ethz.ch/protter/start/). PLXNA1, plexin A1; PLXNA3, plexin A3; PTK7, protein tyrosine kinase 7; CYB5R1, cytochrome b5 reductase 1; FMR1, fragile X messenger ribonucleoprotein 1; GPX7, glutathione peroxidase 7; PTMs, post-translational modifications; TMRs, transmembrane regions.

Figure S8 Representative images of triple immunofluorescence staining for PLXNA1 (green), IBA1 (rose red), and GFAP (blue) in the Cornu Ammonis 1 (CA1) and CA3 region of the hippocampus of young and old mice. Cell nuclei were stained with DAPI. Scale bar = 40 μm. PLXNA1, plexin A1; IBA1, ionized calcium-binding adapter molecule 1; GFAP, glial fibrillary acidic protein.

Figure S1

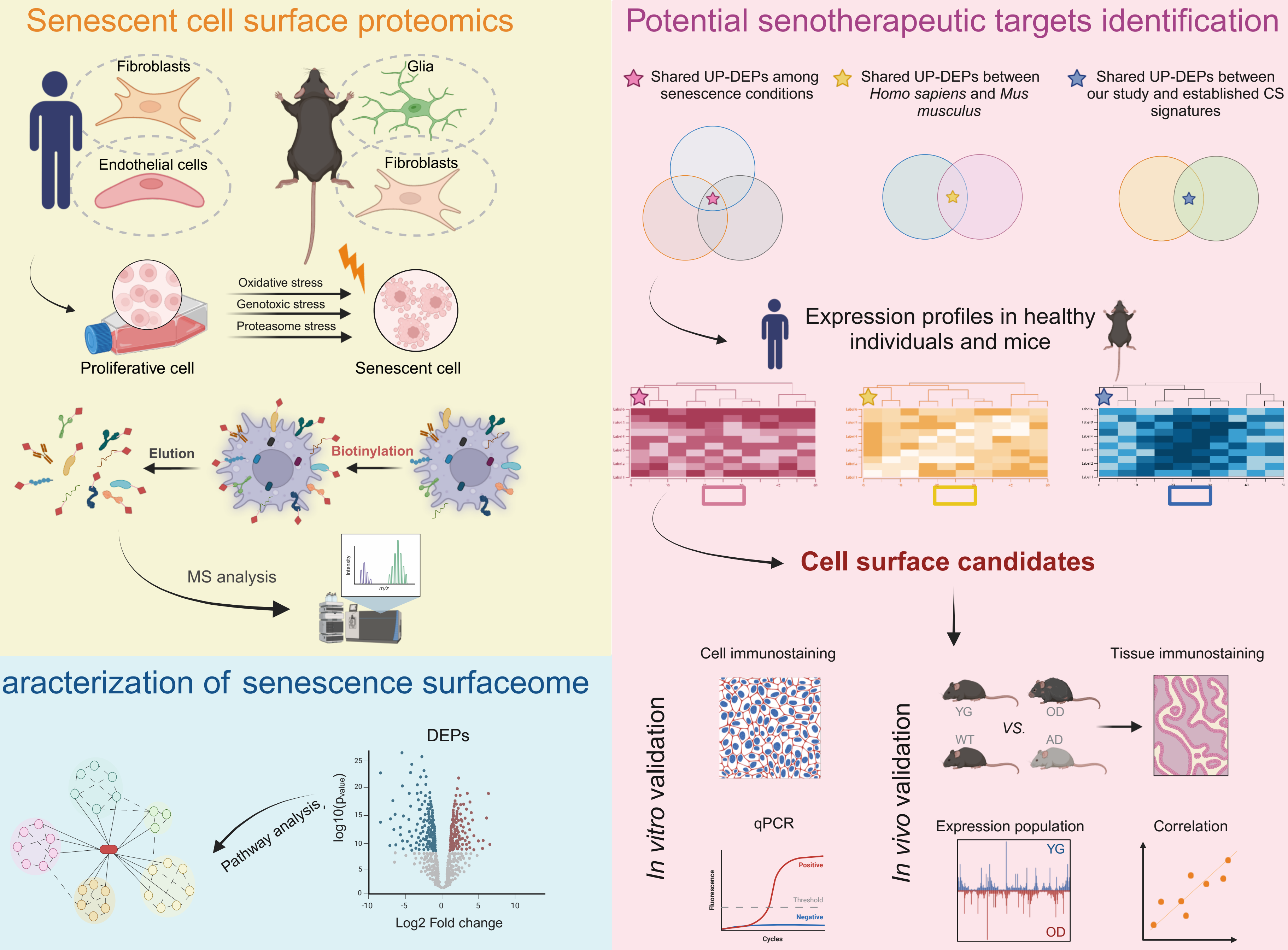

Senescent cell surface proteomics
Fibroblasts
Glia
Endothelial cells
Fibroblasts
Oxidative stress
Genotoxic stress
Proteasome stress
Proliferative cell
Senescent cell
Elution
Biotinylation
MS analysis
Intensity
m/z
Characterization of senescence surfaceome
DEPs
Pathway analysis
-log10(pvalue)
Log2 Fold change
Potential senotherapeutic targets identification
Shared UP-DEPs among senescence conditions
Shared UP-DEPs between Homo sapiens and Mus musculus
Shared UP-DEPs between our study and established CS signatures
Expression profiles in healthy individuals and mice
Cell surface candidates
In vitro validation
Cell immunostaining
qPCR
Positive
Threshold
Negative
Fluorescence
Cycles
In vivo validation
YG
OD
VS.
WT
AD
Tissue immunostaining
Expression population
YG
OD
Correlation

Figure S2

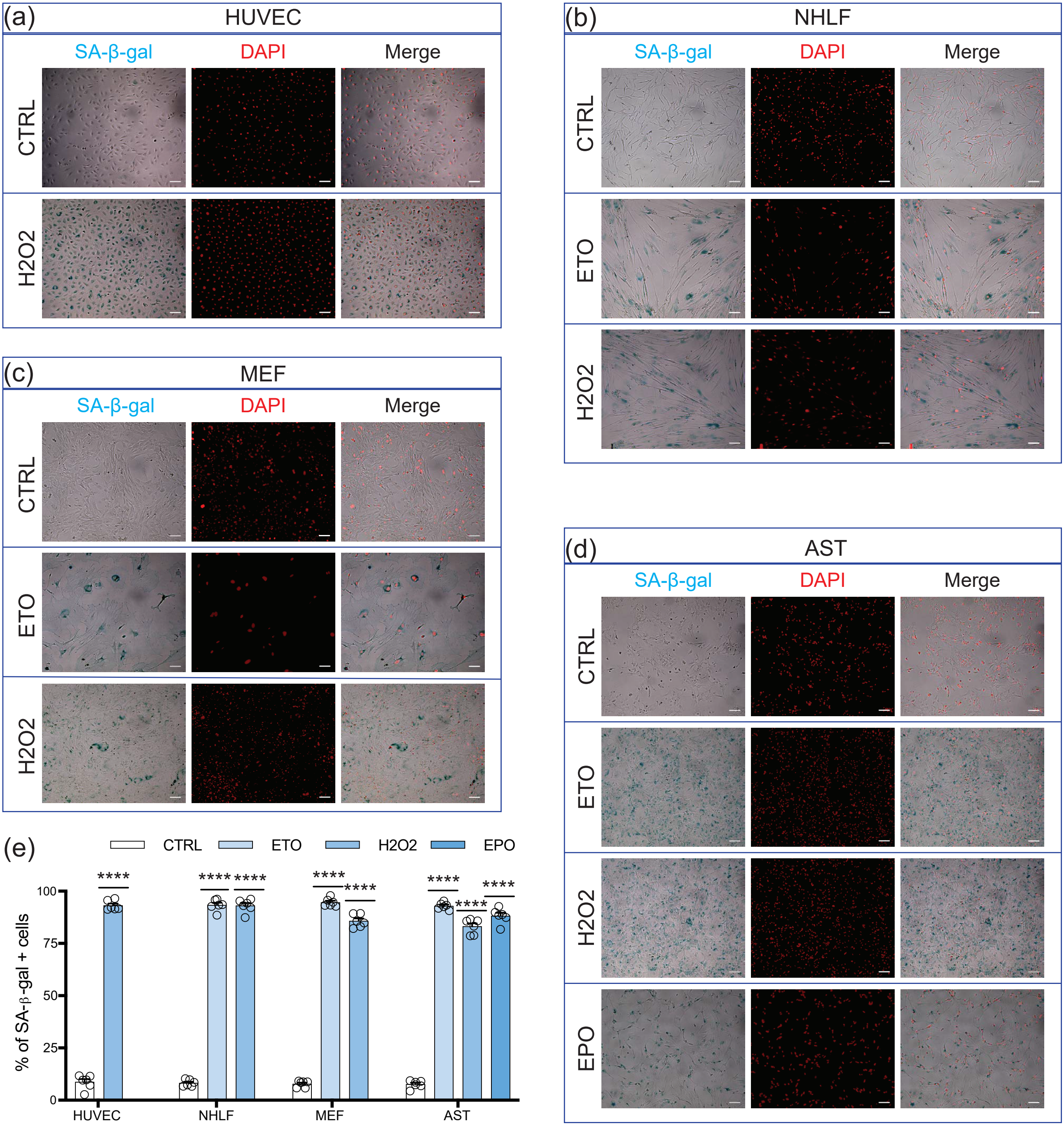

(a)
HUVEC
SA-β-gal
DAPI
Merge
CTRL
H2O2
(b)
NHLF
SA-β-gal
DAPI
Merge
CTRL
ETO
H2O2
(c)
MEF
SA-β-gal
DAPI
Merge
CTRL
ETO
H2O2
(d)
AST
SA-β-gal
DAPI
Merge
CTRL
ETO
H2O2
EPO
(e)
CTRL
ETO
H2O2
EPO
% of SA-β-gal + cells
100
75
50
25
0
****
****
****
****
****
****
****
****
HUVEC
NHLF
MEF
AST

Figure S3

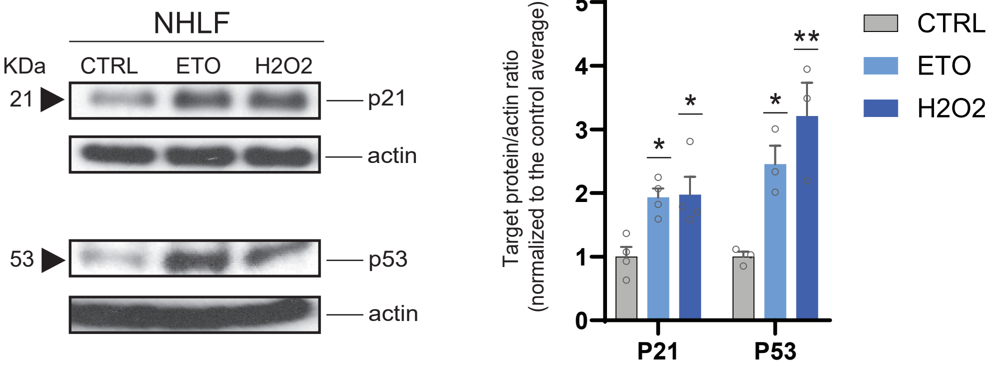
NHLF
KDa
CTRL
ETO
H2O2
21
p21
actin
53
p53
actin
Target protein/actin ratio
(normalized to the control average)
0
1
2
3
4
5
*
*
*
**
P21
P53
CTRL
ETO
H2O2

Figure S4

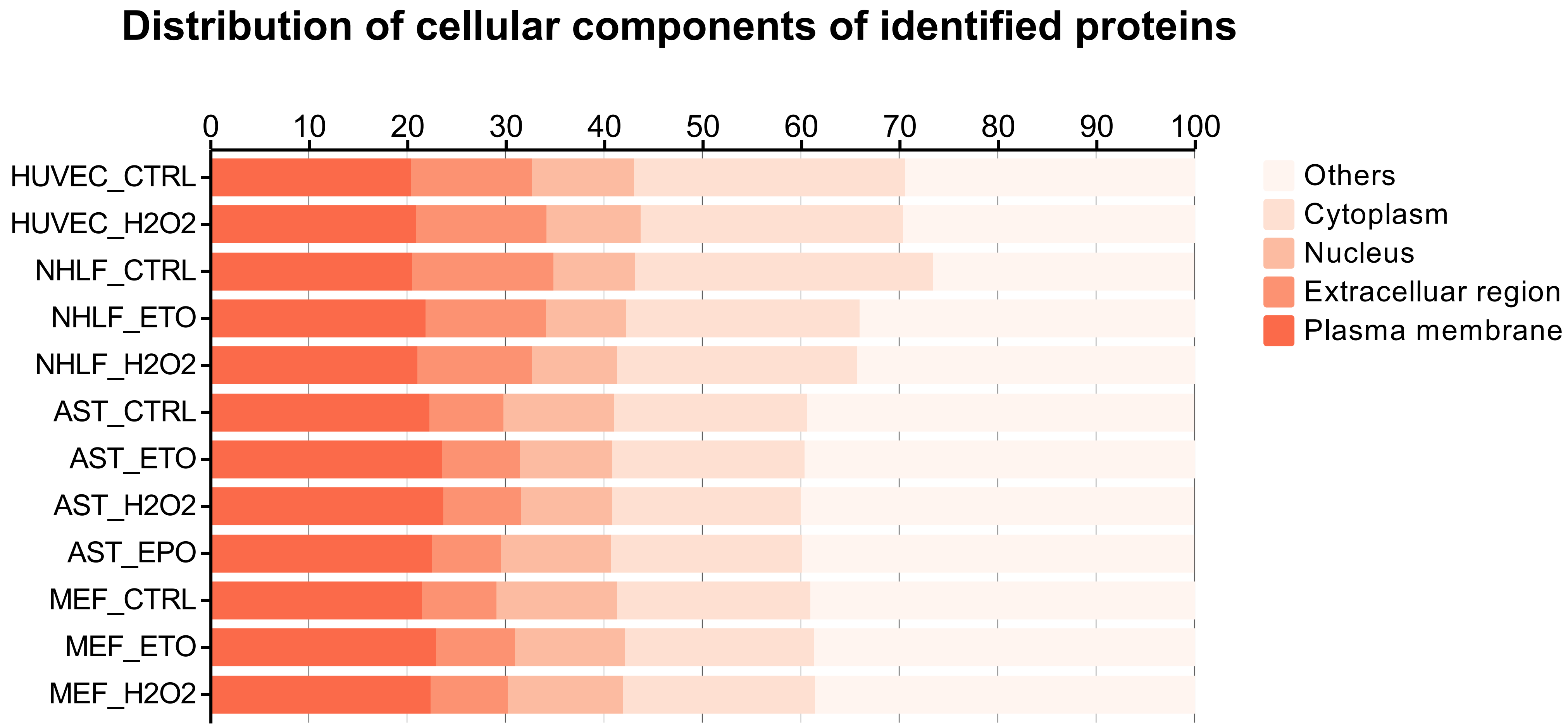
Distribution of cellular components of identified proteins
0
10
20
30
40
50
60
70
80
90
100
HUVEC_CTRL
HUVEC_H2O2
NHLF_CTRL
NHLF_ETO
NHLF_H2O2
AST_CTRL
AST_ETO
AST_H2O2
AST_EPO
MEF_CTRL
MEF_ETO
MEF_H2O2
Others
Cytoplasm
Nucleus
Extracelluar region
Plasma membrane

Figure S5

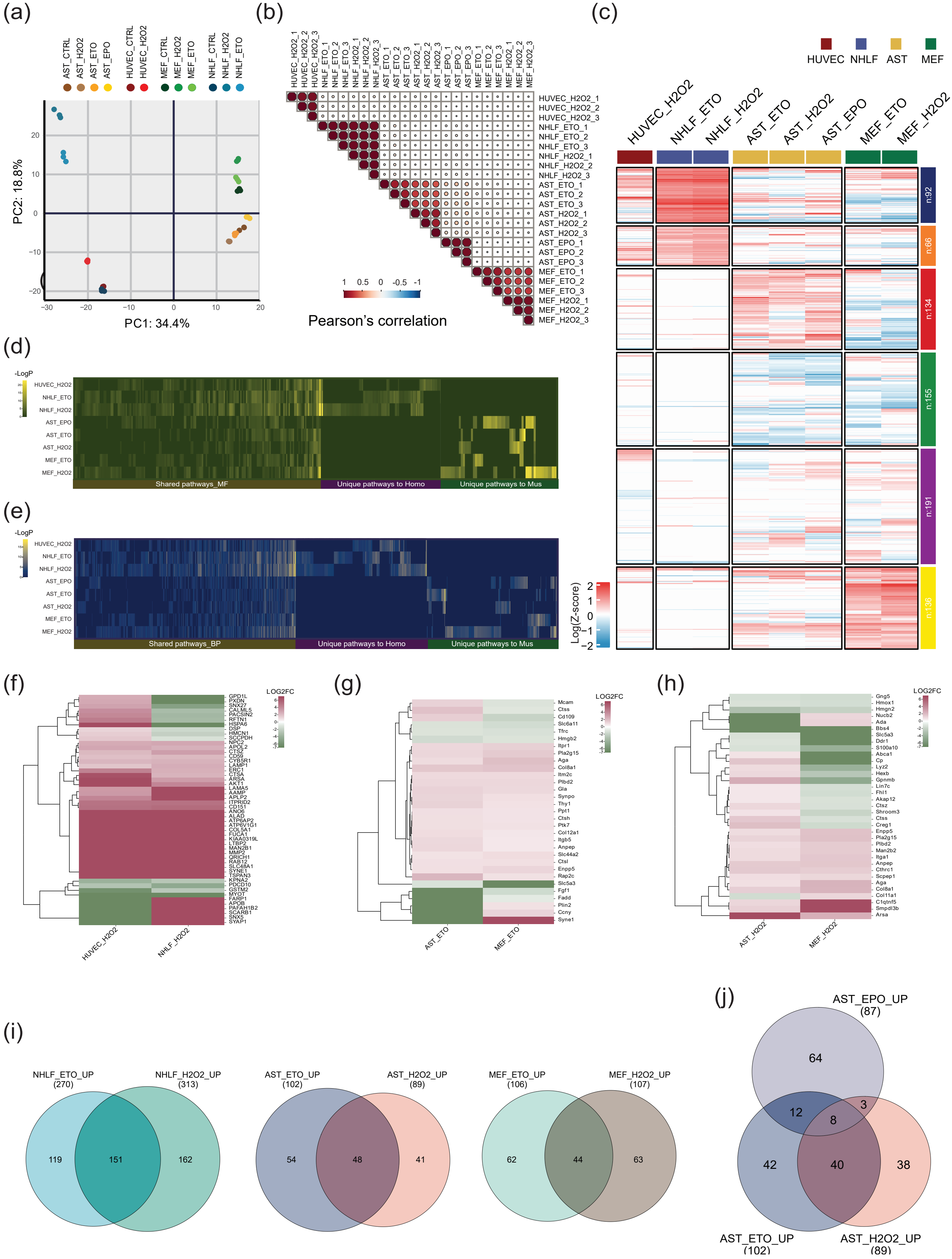

(a)
AST_CTRL
AST_H2O2
AST_ETO
AST_EPO
HUVEC_CTRL
HUVEC_H2O2
MEF_CTRL
MEF_H2O2
MEF_ETO
NHLF_CTRL
NHLF_H2O2
NHLF_ETO
PC2: 18.8%
PC1: 34.4%
(b)
HUVEC_H2O2_1
HUVEC_H2O2_2
HUVEC_H2O2_3
NHLF_ETO_1
NHLF_ETO_2
NHLF_ETO_3
NHLF_H2O2_1
NHLF_H2O2_2
NHLF_H2O2_3
AST_ETO_1
AST_ETO_2
AST_ETO_3
AST_H2O2_1
AST_H2O2_2
AST_H2O2_3
AST_EPO_1
AST_EPO_2
AST_EPO_3
MEF_ETO_1
MEF_ETO_2
MEF_ETO_3
MEF_H2O2_1
MEF_H2O2_2
MEF_H2O2_3
1 0.5 0 -0.5 -1
Pearson's correlation
(c)
HUVEC NHLF AST MEF
HUVEC_H2O2
NHLF_ETO
NHLF_H2O2
AST_ETO
AST_H2O2
AST_EPO
MEF_ETO
MEF_H2O2
n:92
n:66
n:134
n:155
n:191
n:136
Log(Z-score)
2 1 0 -1 -2
(d)
-LogP
HUVEC_H2O2
NHLF_ETO
NHLF_H2O2
AST_EPO
AST_ETO
AST_H2O2
MEF_ETO
MEF_H2O2
Shared pathways_MF
Unique pathways to Homo
Unique pathways to Mus
(e)
-LogP
HUVEC_H2O2
NHLF_ETO
NHLF_H2O2
AST_EPO
AST_ETO
AST_H2O2
MEF_ETO
MEF_H2O2
Shared pathways_BP
Unique pathways to Homo
Unique pathways to Mus
(f)
LOG2FC
HUVEC_H2O2
NHLF_H2O2
(g)
LOG2FC
Mcam
Ctss
Cd109
Slc6a11
Tfrc
Hmgb2
Itpr1
Pla2g15
Aga
Col8a1
Itm2c
Plbd2
Gla
Synpo
Thy1
Ppt1
Ctsh
Ptk7
Col12a1
Itgb5
Anpep
Slc44a2
Ctsl
Enpp5
Rap2c
Slc5a3
Fgf1
Fadd
Plin2
Ccny
Syne1
AST_ETO
MEF_ETO
(h)
LOG2FC
Gng5
Hmox1
Hmgn2
Nucb2
Ada
Bbs4
Slc5a3
Ddr1
S100a10
Abca1
Cp
Lyz2
Hexb
Gpnmb
Lin7c
Fhl1
Akap12
Ctsz
Shroom3
Ctss
Creg1
Enpp5
Pla2g15
Plbd2
Man2b2
Itga1
Anpep
Cthrc1
Scpep1
Aga
Col8a1
Col11a1
C1qtnf5
Smpdl3b
Arsa
AST_H2O2
MEF_H2O2
(i)
NHLF_ETO_UP (270)
NHLF_H2O2_UP (313)
119
151
162
AST_ETO_UP (102)
AST_H2O2_UP (89)
54
48
41
MEF_ETO_UP (106)
MEF_H2O2_UP (107)
62
44
63
(j)
AST_EPO_UP (87)
64
12
3
8
42
40
38
AST_ETO_UP (102)
AST_H2O2_UP (89)

Figure S6

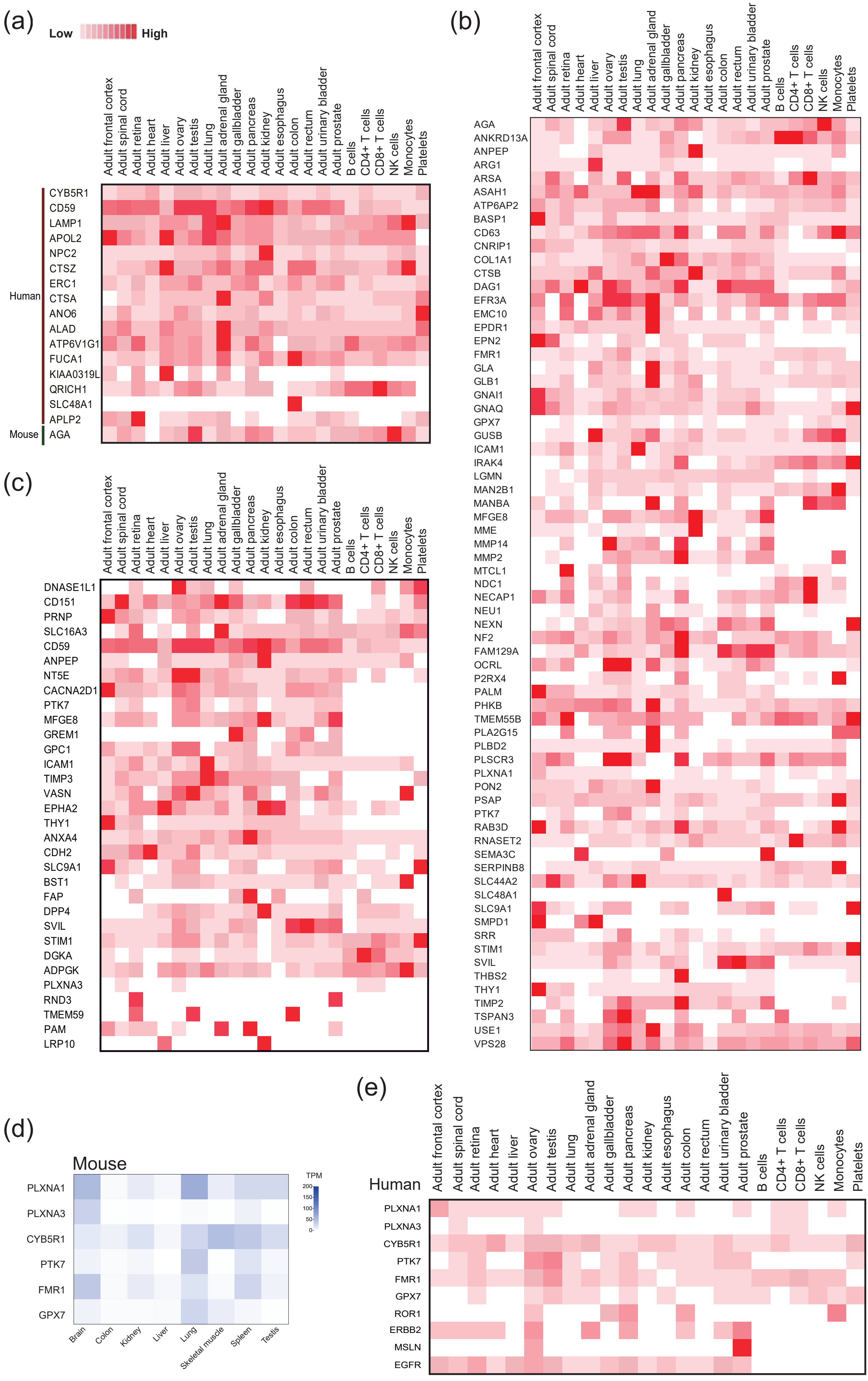
(a)
Low
High
Adult frontal cortex
Adult spinal cord
Adult retina
Adult heart
Adult liver
Adult ovary
Adult testis
Adult lung
Adult adrenal gland
Adult gallbladder
Adult pancreas
Adult kidney
Adult esophagus
Adult colon
Adult rectum
Adult urinary bladder
Adult prostate
B cells
CD4+ T cells
CD8+ T cells
NK cells
Monocytes
Platelets
Human
CYB5R1
CD59
LAMP1
APOL2
NPC2
CTSZ
ERC1
CTSA
ANO6
ALAD
ATP6V1G1
FUCA1
KIAA0319L
QRICH1
SLC48A1
APLP2
Mouse
AGA
(b)
AGA
ANKRD13A
ANPEP
ARG1
ARSA
ASAH1
ATP6AP2
BASP1
CD63
CNRIP1
COL1A1
CTSB
DAG1
EFR3A
EMC10
EPDR1
EPN2
FMR1
GLA
GLB1
GNAI1
GNAQ
GPX7
GUSB
ICAM1
IRAK4
LGMN
MAN2B1
MANBA
MFGE8
MME
MMP14
MMP2
MTCL1
NDC1
NECAP1
NEU1
NEXN
NF2
FAM129A
OCRL
P2RX4
PALM
PHKB
TMEM55B
PLA2G15
PLBD2
PLSCR3
PLXNA1
PON2
PSAP
PTK7
RAB3D
RNASET2
SEMA3C
SERPINB8
SLC44A2
SLC48A1
SLC9A1
SMPD1
SRR
STIM1
SVIL
THBS2
THY1
TIMP2
TSPAN3
USE1
VPS28
(c)
DNASE1L1
CD151
PRNP
SLC16A3
CD59
ANPEP
NT5E
CACNA2D1
PTK7
MFGE8
GREM1
GPC1
ICAM1
TIMP3
VASN
EPHA2
THY1
ANXA4
CDH2
SLC9A1
BST1
FAP
DPP4
SVIL
STIM1
DGKA
ADPGK
PLXNA3
RND3
TMEM59
PAM
LRP10
(d)
Mouse
TPM
200
150
100
50
0
PLXNA1
PLXNA3
CYB5R1
PTK7
FMR1
GPX7
Brain
Colon
Kidney
Liver
Lung
Skeletal muscle
Spleen
Testis
(e)
Human
PLXNA1
PLXNA3
CYB5R1
PTK7
FMR1
GPX7
ROR1
ERBB2
MSLN
EGFR

Figure S7

Schematic visualization of six potential senotherapeutic targets

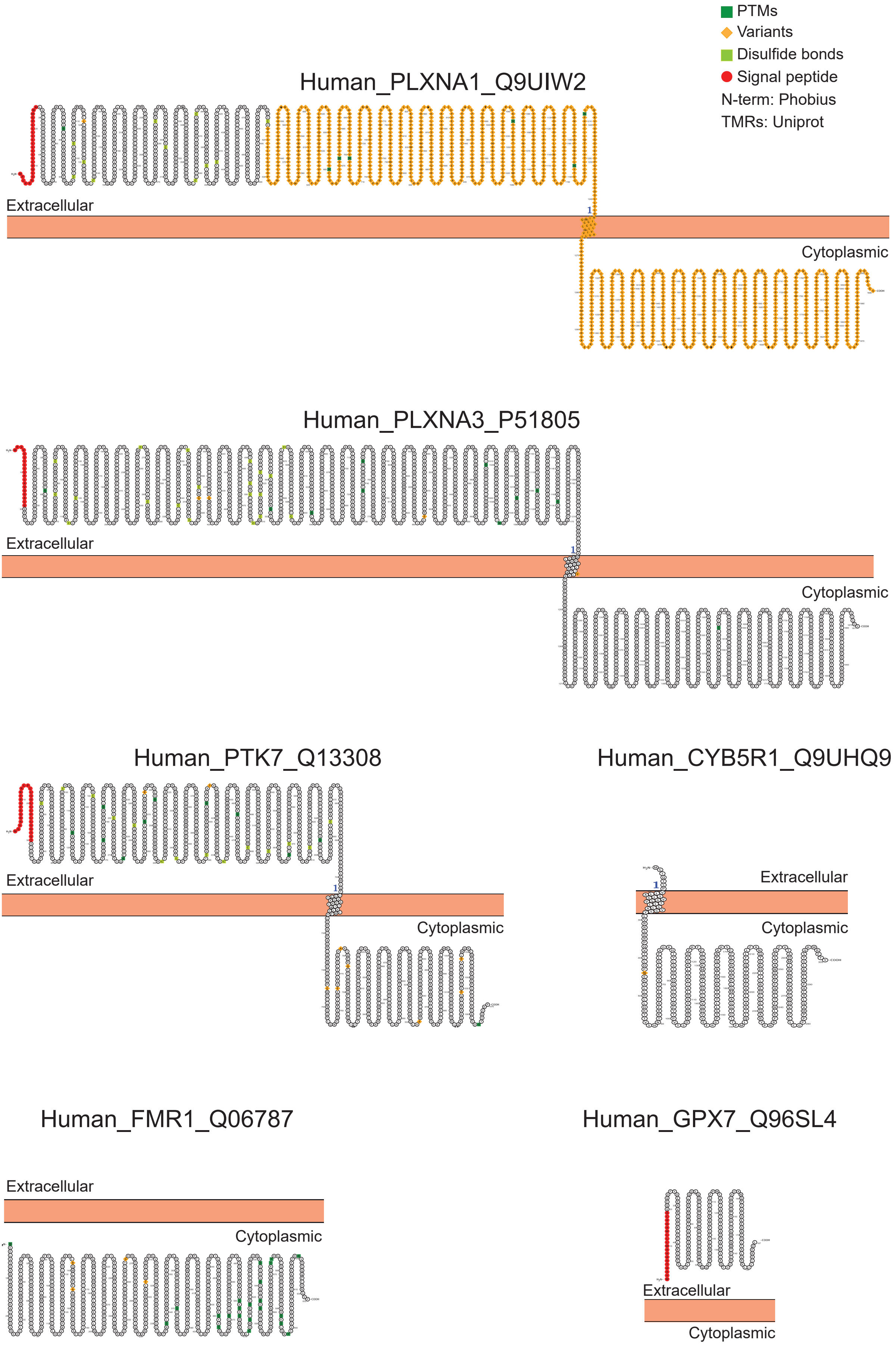

Figure S8

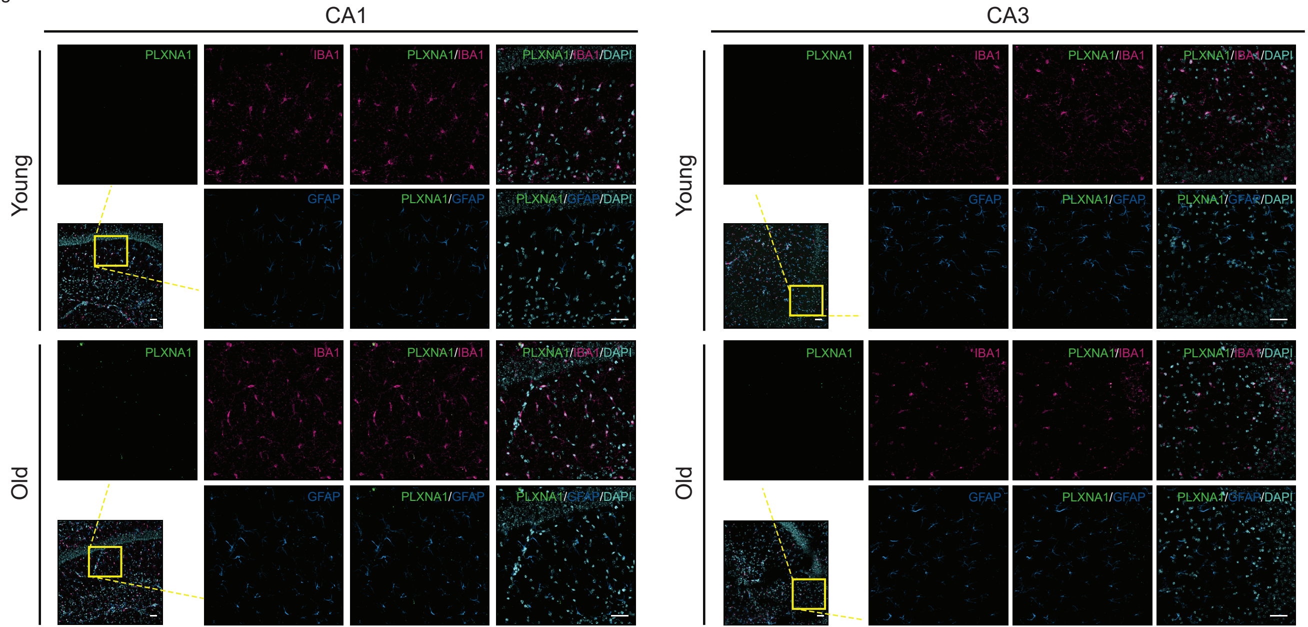
CA1
CA3
Young
Old
PLXNA1
IBA1
PLXNA1/IBA1
PLXNA1/IBA1/DAPI
GFAP
PLXNA1/GFAP
PLXNA1/GFAP/DAPI
